# Gold(I) Complexes Bearing Alkylated 1,3,5-Triaza-7-phosphaadamantane Ligands as Thermoresponsive Anticancer Agents in Human Colon Cells

**DOI:** 10.3390/biomedicines9121848

**Published:** 2021-12-06

**Authors:** Javier Quero, Francesco Ruighi, Jesús Osada, M. Concepción Gimeno, Elena Cerrada, Maria Jesús Rodriguez-Yoldi

**Affiliations:** 1 Departamento de Química Inorgánica, Instituto de Síntesis Química y Catálisis Homogénea-ISQCH, Universidad de Zaragoza-C.S.I.C., 50009 Zaragoza, Spain; javierquero94@gmail.com (J.Q.); francesco.ruighi@edu.unito.it (F.R.); gimeno@unizar.es (M.C.G.); 2 CIBERobn, IIS Aragón, IA2, Departamento de Farmacología y Fisiología y Medicina Legal y Forense, Unidad de Fisiología, Universidad de Zaragoza, 50013 Zaragoza, Spain; 3 CIBERobn, IIS Aragón, IA2, Departamento de Bioquímica y Biología Molecular, Unidad de Bioquímica, Universidad de Zaragoza, 50013 Zaragoza, Spain; josadal@unizar.es

**Keywords:** hyperthermia, PTA, gold, thiolate, colon cancer, TrxR

## Abstract

Overheating can affect solubility or lipophilicity, among other properties, of some anticancer drugs. These temperature-dependent changes can improve efficiency and selectivity of the drugs, since they may affect their bioavailability, diffusion through cell membrane or activity. One recent approach to create thermosensitive molecules is the incorporation of fluorine atoms in the chemical structure, since fluor can tune some chemical properties such as binding affinity. Herein we report the anticancer effect of gold derivatives with phosphanes derived from 1,3,5-triaza-7-phosphaadamantane (PTA) with long hydrocarbon chains and the homologous fluorinated chains. Besides, we analysed the influence of temperature in the cytotoxic effect. The studied gold(I) complexes with phosphanes derived from PTA showed antiproliferative effect on human colon carcinoma cells (Caco-2/TC7 cell line), probably by inhibiting cellular TrxR causing a dysfunction in the intracellular redox state. In addition, the cell cycle was altered by the activation of p53, and the complexes produce apoptosis through mitochondrial depolarization and the consequent activation of caspase-3. Furthermore, the results suggest that this cytotoxic effect is enhanced by hyperthermia and the presence of polyfluorinated chains.

## 1. Introduction

Mitochondria have emerged as important biological targets, since they are known as an essential biosynthetic, bioenergetic, and signalling organelles, playing a key role in cellular differentiation, proliferation, and death [1]. Mitochondria also play a central and multifunctional role in malignant tumour progression. Therefore, cancer has been recently considered as a mitochondrial metabolic disease [2] and consequently, targeting mitochondria provides new therapeutic opportunities. Anticancer agents that directly target mitochondria do not exert their mode of action via interaction with nuclear DNA. Consequently, these drugs are likely to be minimally genotoxic.

There are many examples of compounds with anticancer activity that act on mitochondria. The non-metal anti-mitochondrial agents, named as ‘mitocans’ as the acronym for ‘mitochondria and cancer’ [3], have been classified into eight classes depending on the mitochondrial site of action. Compounds targeting Bcl-2 family proteins, thiol redox inhibitors and VDAC/ANT targeting compounds, electron redox chain targeting drugs and lipophilic cations among others constitute a vast group of promising anti-carcinogenic drugs [3,4]. In addition, a wide number of cytotoxic metallodrugs, whose mechanism of action is based on to their ability to alter mitochondrial function, have been described [5].

Most of the anti-mitochondrial metal complexes are Au-, Ru-, Ir- and Pt-based compounds and exert their activity via mitochondrial damage and mitochondria-mediated apoptosis, thioredoxin reductase inhibition and/or interaction with protein translocators. Gold derivatives are probably the best studied anticancer metal complexes that target mitochondria. Several lines of evidence point that the enzyme thioredoxin reductase (TrxR) constitutes the main target for cytotoxic gold derivatives [6,7,8,9]. The presence of cysteine and selenocysteine residues in the catalytic sites of the enzyme can bind soft metals such as gold, with a subsequent inhibition of its activity. TrxR, a ubiquitous flavoprotein that maintains the cellular redox state [10], is existing as two main isoforms, namely, the cytosolic (TrxR1) and the mitochondrial (TrxR2). Both isoforms exert the same function of reducing the disulfide protein thioredoxin to its dithiol form. Its inhibition affects the cellular redox balance by increasing ROS levels leading to a decrease in the mitochondrial thiol levels, which consequent can affect the mitochondrial membrane permeability causing apoptosis [11]. Examples that include phosphane thiolate [12,13,14] and phosphane alkynyl gold(I) derivatives [15,16,17,18], NHC-carbene gold(I) complexes [19,20,21,22], cationic bisphosphane complexes [23] or square-planar gold(III) compounds [24] have been described as TrxR inhibitors.

Tumor cells exhibit a more negative mitochondrial transmembrane potential in comparison with normal cells [25], that contributes to enhanced accumulation to positively charged compounds, such as lipophilic cationic metal complexes. Among them, cationic Au(I)-N-heterocyclic carbene complexes constitute one of the most studied groups of mitochondria-targeting lipophilic cationic metal derivatives. However, the accumulation in mitochondria of type of lipophilic species may not be selective towards cancerous cells, and the side effects may be significant.

Recent studies have revealed that the temperature of mitochondria is higher than the ambient temperature and estimated around 10 degrees higher [26]. Taking advantage of that temperature difference, some studies related to thermoresponsive drug delivery have been recently described [27,28]. Besides, thermoresponsive small molecules [29], such as arene ruthenium complexes [30,31], platinum compounds [32] and chlorambucil derivatives [33], both with long polyfluorinated appendages, have shown an increased cytotoxic activity under hyperthermia conditions.

With this idea we describe here the synthesis of gold derivatives with phosphanes derived from 1,3,5-triaza-7-phosphaadamantane (PTA) after introduction of perfluorinated chains in order to lead to thermoresponsive properties. Related compounds with long hydrocarbon chains in the PTA skeleton, were also included for comparison purposes. The new complexes were tested against human colon carcinoma cells (Caco-2 cells) at 37 °C and following a hyperthermia treatment at 40 °C and their possible intracellular target was also studied, including TrxR and possible mitochondrial disturbances and apoptotic implications.

## 2. Materials and Methods

### 2.1. Synthesis of the Ligands

**Synthesis of [PTA-CH_2_(CH_2_)_6_CH_3_]I (La).** To a solution of PTA (1 mmol, 0.157 g) in acetone under argon atmosphere, 1-Iodooctane (1 mmol) was added. The resulting suspension was stirred for 3 days. The white product obtained was then filtered, washed with cold acetone and diethyl ether and dried under vacuum. The product was isolated as a white solid (yield 90%).

^1^H NMR (400 MHz, dmso-d_6_, 25 °C): δ = 4.96 and 4.79 (AB system, J_AB_ = 11.2 Hz, 4H, N^+^CH_2_N), 4.52 and 4.33 (AB system, J_AB_ = 12.0 Hz, 2H, NCH_2_N), 4.34 (m, 2H, N^+^CH_2_P), 3.93 (t, J = 13.7 Hz, 2H, NCH_2_P), 3.82 (m, 2H, NCH_2_P), 2.79 (m, 2H, N^+^CH_2_R), 1.67 (m, 2H, CH_2_), 1.29 (m, 10H, CH_2_), 0.87 (t, J = 6.8 Hz, 3H, CH_3_) ppm. ^31^P{^1^H} NMR (162 MHz, dmso-d_6_, 25 °C): δ = −85.7 (s) ppm. ^13^C{^1^H} NMR (101 MHz, dmso-d_6_, 25 °C): δ = 79.0 (s, 2C, N^+^CH_2_N), 69.8 (s, 1C, NCH_2_N), 61.8 (s, 1C, N^+^CH_2_R), 52.2 (d, J = 31.9 Hz, 1C, N^+^CH_2_P), 45.9 (d, J = 20.3 Hz, 2C, NCH_2_P), 31.7 (s, 1C, C_intrachain_), 28.9 (s, 2C, C_intrachain_), 26.6 (s, 1C, C_intrachain_), 22.5 (s, 1C, C_intrachain_), 19.5 (s, 1C, N^+^CH_2_CH_2_R), 14.4 (s, 1C, CH_3_) ppm. IR ν_max_/cm^−1^: 2923 (m, stretching C-H), 1465 and 1414 and 1370 (m, bending -C-H). ESI *m*/*z* (%): 269.9 [M]^+^ (100). Anal. calcd. (%) for C_14_H_29_IN_3_P (397.27): C, 42.33; H, 7.36; N, 10.58. Found: C, 42.13; H, 7.16; N, 10.75.

**Synthesis of TfOCH_2_CH_2_(CF_2_)_5_CF_3_**. To a solution of 1H,1H,2H,2H-Perfluoro-1-octanol (5 mmol) and pyridine (5 mmol) in 10 mL of dry dichloromethane under argon atmosphere and cooled to −78 °C, trifluoromethanesulfonic anhydride (7.5 mmol) was added dropwise. The resulting solution was stirred at −78 °C for 35 min. Then the reaction was allowed to warm to room temperature and stirred for 3 h. Then, the reaction was quenched with water (10 mL) and dichloromethane (10 mL). The aqueous phase was extracted with dichloromethane (2 × 10 mL). The organic phase was dried with MgSO_4_ and the solvent evaporated. The crude organic residue was eluted with dichloromethane on silica gel to afford red-brown oil (74% yield).

^1^H NMR (400 MHz, CDCl_3_, 25 °C): δ = 4.77 (t, J = 6.3 Hz, 2H, CH_2_-O-), 2.66 (tt, J = 17.4 and 6.2 Hz, 2H, CH_2_-CF_2_) ppm. ^19^F{^1^H} NMR (376 MHz, CDCl_3_, 25 °C): δ = −75.1 (s, TfO^−^), −81.29 (tt, J = 10.1 and 2.3 Hz, CF_3_), −113.9 (m, CF_2_), −122.1 (m, CF_2_), −123.2 (s, CF_2_), −123.8 (m, CF_2_), −126.5 (m, CF_2_) ppm.

**Synthesis of [PTA-CH_2_CH_2_(CF_2_)_5_CF_3_]TfO (Lb).** To a cooled solution (0 °C) of PTA (1 mmol, 0.157 g) in acetone under argon atmosphere, TfOCH_2_CH_2_(CF_2_)_5_CF_3_ (1 mmol, 0.496 g) was added dropwise and the resulting solution was stirred for 35 min at 0 °C. The solution was allowed to warm to room temperature and then evaporated. The remaining solution was precipitated with diethyl ether. The white solid resulting was filtered and washed with cold acetone and diethyl ether (89% yield).

^1^H NMR (400 MHz, dmso-d_6_, 25 °C): δ = 5.08 and 4.90 (AB system, J_AB_ = 10.8 Hz, 4H, N^+^CH_2_N), 4.54 and 4.32 (AB system, J_AB_ = 13.2 Hz, 2H, NCH_2_N), 4.42 (d, J = 5.2 Hz, 2H, N^+^CH_2_P), 3.96 (t, J = 13.7 Hz, 2H, NCH_2_P), 3.80 (dd, J = 14.4, 8.5 Hz, 2H, NCH_2_P), 3.20 (m, 2H, N^+^CH_2_), 2.93 (m, 2H, CH_2_CF_2_) ppm. ^31^P{^1^H} NMR (162 MHz, dmso-d_6_, 25 °C): δ = −85.0 (s) ppm. ^13^C{^1^H} NMR (101 MHz, dmso-d_6_, 25 °C): δ = 121.3–108.2 (m series, 6C, -CH_2_-CF_2_-CF_2_-, -CH_2_-CF_2_-CF_2_-CF_2_-, -CH_2_-(CF_2_)_2_-CF_2_-, -CH_2_-(CF_2_)_3_-CF_2_-, -CH_2_-(CF_2_)_4_-CF_2_-CF_3_), 79.3 (s, 2C, N^+^CH_2_N), 69.2 (s, 1C, NCH_2_N), 52.7 (s, 1C, N^+^CH_2_R), 51.8 (d, J = 32.7 Hz, 1C, N^+^CH_2_P), 45.4 (d, J = 20.5 Hz, 2C, NCH_2_P), 21.8 (t, J_CF_ = 20.9 Hz, 1C, N^+^CH_2_CH_2_R) ppm. ^19^F{^1^H} NMR (376 MHz, dmso-d_6_, 25 °C): δ = −77.8 (s, TfO^−^), −80.2 (t, J = 8.6 Hz, CF_3_), −112.8 (m), −121.7 (s, CF_2_), −122.4 (s, CF_2_), −122.6 (s, CF_2_), −125.8 (s, CF_2_) ppm. IR ν_max_/cm^−1^: 3004 and 2916 (w, stretching C-H), 1467 and 1420 (w, bending -C-H), 1224 (s, stretching C-F), 1142 (s, asymmetric stretching C-F). MALDI MS *m*/*z* (%): 504.1 [M]^+^ (100). Anal. calcd. (%) for C_15_H_16_F_16_N_3_O_3_PS (653.04): C, 27.58; H, 2.47; N, 6.43; S, 4.91. Found: C, 27.69; H, 2.76; N, 6.72; S, 5.30.

### 2.2. Synthesis of the Gold Complexes: [AuCl(PTA-R)]X

To a solution of [AuCl(tht)] (0.5 mmol, 0.160 g) in dichloromethane (15 mL), the alkylated PTA **La** or **Lb** (0.5 mmol) was added. The resulting suspension was stirred for 4–5 h at room temperature. The solid formed in the round-bottom flask was filtered and washed with cold acetone (5 mL) and diethyl ether (10 mL) to eliminate all the tetrahydrothiophene. The white solid was then dried in air and stored at 5 °C.

### 2.3. Synthesis of Gold Complexes [AuCl(PTA-R)_2_]X_2_

To a suspension of [AuCl(PTA-CH_2_-(CH_2_)_6_-CH_3_)]I (0.15 mmol, 0.095 g) or [AuCl(PTA-CH_2_-CH_2_-(CF_2_)_5_-CF_3_)]TfO (0.15 mmol, 0.133 g) in acetone (15 mL) the corresponding alkylated PTA (La or Lb) (0.15 mmol) was added. The suspension was stirred for 5 h at room temperature. The resulting white solid was filtered, washed with diethyl ether and dried in air and finally stored at 5 °C.

### 2.4. Synthesis of Gold Complexes [Au(R’S)(PTA-R)]X

To a solution of 2-mercaptopyridine (0.20 mmol) in ethanol under argon atmosphere, NaOEt was added (0.22 mmol). The solution was stirred for 15 min. Maintaining inert atmosphere, [AuCl(tht)] (0.20 mmol, 0.064 g) was added and the suspension formed was stirred for 2 h. Then the resulting solid and the solution were separated by removing the liquid phase. The solid, settled on the bottom of the schlenk, was washed two times with ethanol. After removal of ethanol, the corresponding alkylated PTA (**La** or **Lb**) was added in acetone (15mL). The mixture was stirred overnight, and the solution was evaporated, precipitated with diethyl ether, and filtered. The resulting solid was dried in air and stored at low 5 °C.

The following characterization was carried out to confirm the synthesis of the new gold complexes: 

**[AuCl(PTA-CH_2_(CH_2_)_6_CH_3_)]I (1a)**. Yield: 90%, white solid. ^1^H NMR (400 MHz, dmso-d_6_, 25 °C): δ= 5.08 and 4.90 (AB system, J_AB_ = 10.8 Hz, 4H, N^+^CH_2_N), 4.54 and 4.32 (AB system, J_AB_ = 13.2 Hz, 2H, NCH_2_N), 4.42 (d, J = 5.2 Hz, 2H, N^+^CH_2_P), 3.96 (t, J = 13.7 Hz, 2H, NCH_2_P), 3.80 (dd, J = 14.4, 8.5 Hz, 2H, NCH_2_P), 3.20 (m, 2H, N^+^CH_2_), 2.93 (m, 2H, CH_2_CF_2_) ppm. ^31^P{^1^H} NMR (162 MHz, dmso-d_6_, 25 °C): δ = −85.0 (s) ppm. ^13^C{^1^H} NMR (101 MHz, dmso-d_6_, 25 °C): δ = 121.3–108.2 (m series, 6C, -CH_2_-CF_2_-CF_2_-, -CH_2_-CF_2_-CF_2_-CF_2_-, -CH_2_-(CF_2_)_2_-CF_2_-, -CH_2_-(CF_2_)_3_-CF_2_-, -CH_2_-(CF_2_)_4_-CF_2_-CF_3_), 79.3 (s, 2C, N^+^CH_2_N), 69.2 (s, 1C, NCH_2_N), 52.7 (s, 1C, N^+^CH_2_R), 51.8 (d, J = 32.7 Hz, 1C, N^+^CH_2_P), 45.4 (d, J = 20.5 Hz, 2C, NCH_2_P), 21.8 (t, J_CF_ = 20.9 Hz, 1C, N^+^CH_2_CH_2_R) ppm. ^19^F{^1^H} NMR (376 MHz, dmso-d_6_, 25 °C): δ = −77.8 (s, TfO^−^), −80.2 (t, J = 8.6 Hz, CF_3_), −112.8 (m), −121.7 (s, CF_2_), −122.4 (s, CF_2_), −122.6 (s, CF_2_), −125.8 (s, CF_2_) ppm. IR ν_max_/cm^−1^: 3004 and 2916 (w, stretching C-H), 1467 and 1420 (w, bending -C-H), 1224 (s, stretching C-F), 1142 (s, asymmetric stretching C-F). MALDI MS *m*/*z* (%): 504.1 [M]^+^ (100). Anal. calcd. (%) for C_15_H_16_F_16_N_3_O_3_PS (653.04): C, 27.58; H, 2.47; N, 6.43; S, 4.91. Found: C, 27.69; H, 2.76; N, 6.72; S, 5.30.

**[AuCl(PTA-CH_2_CH_2_(CF_2_)_5_CF_3_)]TfO(1b)**. Yield: 93%, white solid. ^1^H NMR (400 MHz, dmso-d_6_, 25 °C): δ = 5.09 and 4.88 (AB system, J_AB_ = 11.6 Hz, 4H, N^+^CH_2_N), 4.75 (d, J *=* 5.2 Hz, 2H, N^+^CH_2_P), 4.61 and 4.53 (AB system, J_AB_ = 14.4 Hz, 2H, NCH_2_N), 4.38 (t, J = 14.2 Hz, 2H, NCH_2_P), 4.24 (m, 2H, NCH_2_P), 2.94 (t, J = 7.4 Hz, 2H, N^+^CH_2_R), 1.67 (m, 2H, CH_2_), 1.27 (m, 10H, CH_2_), 0.86 (t, J = 6.8 Hz, 3H, CH_3_) ppm. ^31^P{^1^H} NMR (162 MHz, dmso-d_6_, 25 °C): δ = −29.8 (s) ppm. ^13^C{^1^H} NMR (101 MHz, dmso-d_6_, 25 °C): δ = 78.3 (s, 2C, N^+^CH_2_N), 68.6 (s, 1C, NCH_2_N), 61.1 (s, 1C, N^+^CH_2_R), 53.0 (d, J = 31.9 Hz, 1C, N^+^CH_2_P), 48.3 (d, J = 20.3 Hz, 2C, NCH_2_P), 31.2 (m, 1C, C_intrachain_), 28.4 (m, 2C, C_intrachain_), 26.1 (s, 1C, C_intrachain_), 22.1 (s, 1C, C_intrachain_), 19.2 (s, 1C, N^+^CH_2_CH_2_R), 14.0 (s, 1C, CH_3_) ppm. IR ν_max_/cm^−1^: 2921 (m, stretching C-H), 1454 and 1413 (m, bending -C-H), 319 (m, stretching Au-Cl). MALDI MS *m*/*z* (%): 502.3 [M]^+^ (58.0). Anal. calcd. (%) for C_14_H_29_AuClIN_3_P (629.69): C, 26.70; H, 4.64; N, 6.67. Found: C, 26.34; H, 5.03; N, 6.66.

**[AuCl(PTA-CH_2_(CH_2_)_6_CH_3_)_2_]I_2_ (2a).** Yield: 60%, white solid. ^1^H NMR (400 MHz, dmso-d_6_, 25 °C): δ = 5.18 and 4.84 (AB system, J_AB_ = 11.2 Hz, 8H, N^+^CH_2_N), 4.67 (s, 4H, N^+^CH_2_P), 4.65–6.40 (AB system, 4H, NCH_2_N), 4.40–3.90 (m, 4H, NCH_2_P), 2.91 (t, J = 7.6 Hz, 4H, N^+^CH_2_R), 1.71 (m, 4H, CH_2_), 1.27 (m, 20H, CH_2_), 0.86 (t, J = 6.8 Hz, 6H, CH_3_) ppm. ^31^P NMR (162 MHz, dmso-d_6_, 25 °C): δ = −57.8 (s) ppm. ^13^C{^1^H} NMR (101 MHz, dmso-d_6_, 25 °C): δ = 81.6 (s, 4C, N^+^CH_2_N), 74.6 (s, 2C, NCH_2_N), 58.6 (s, 2C, N^+^CH_2_R), 48.5 (m, 2C, N^+^CH_2_P), 42.5 (s, 4C, NCH_2_P), 31.7 (s, 2C, C_intrachain_), 29.1 (s, 2C, N^+^CH_2_CH_2_R), 28.7 (s, 2C, C_intrachain_), 25.2 (s, 2C, C_intrachain_), 22.5, (s, 4C, C_intrachain_), 14.5 (s, 2C, CH_3_) ppm. IR ν_max_/cm^−1^: 2923 and 2853 (m, stretching C-H), 1454 and 1417 (m, bending -C-H). Anal. calcd. (%) for C_28_H_58_AuClI_2_N_6_P_2_ (1026.97): C, 32.75; H, 5.69; N, 8.18. Found: C, 33.08; H, 5.93; N, 8.51.

**[AuCl(PTA-CH_2_CH_2_(CF_2_)_5_CF_3_)_2_](TfO)_2_(2b).** Yield: 64%, white solid. ^1^H NMR 206 (400 MHz, dmso-d_6_, 25 °C): δ = 5.16 and 4.94 (AB system, J = 11.6 Hz, 8H, N^+^CH_2_N), 4.58 (m, 4H, N^+^CH_2_P), 4.51 and 4.32 (AB system, J = 12.8 Hz, 4H, NCH_2_N), 4.14 (m, 4H, NCH_2_P), 3.99 (m, 4H, NCH_2_P), 3.30 (m, 4H, N^+^CH_2_R), 2.94 (m, 4H, CH_2_) ppm. ^31^P NMR (162 MHz, dmso-d6, 25 °C): δ = −59.7 ppm. ^13^C{^1^H} NMR (101 MHz, dmso-d_6_, 25 °C): δ = 120.3–107.8 (m series, 12C, -CH_2_-CF_2_-CF_2_-, -CH_2_-CF_2_-CF_2_-CF_2_-, -CH_2_-(CF_2_)_2_-CF_2_-, -CH_2_-(CF_2_)_3_-CF_2_-, -CH_2_-(CF_2_)_4_-CF_2_-CF_3_), 79.5 (s, 4C, N^+^CH_2_N), 68.9 (s, 2C, NCH_2_N), 52.7 (s, 2C, N^+^CH_2_P), 52.7 (s, 2C, N^+^CH_2_R), 47.0 (m, 4C, NCH_2_P), 21.9 (d, J_CF_ = 21.0 Hz, 2C, N^+^CH_2_CH_2_CF_2_) ppm. ^19^F {^1^H} NMR (376 MHz, dmso-d_6_, 25 °C): δ = −77.8 (s, TfO^−^), −80.2 (m, CF_3_), −112.8 (s, CF_2_), −121.7 (s, CF_2_), −122.4 and −122.6 (s, CF_2_), −125.8 (s, CF_2_) ppm. IR ν_max_/cm^−1^: 2997 (w, stretching C-H), 1423 (w, bending -C-H), 1227 (s, stretching C-F), 1144 (s, asymmetric stretching C-F), 325 (w, stretching Au-Cl). MALDI MS *m*/*z* (%): 504.5 [PTA-R]^+^ (100), 736.5 [M-AuCl(PTA-R)]^+^ (32.77). Anal. calcd. (%) for C_30_H_32_AuClF_32_N_6_O_6_P_2_S_2_ (1538.01): C, 23.41; H, 2.10; N, 5.46; S, 4.17. Found: C, 23.58; H, 2.15; N, 5.71; S, 4.53.

**[Au(C_5_H_5_NS)(PTA-CH_2_(CH_2_)_6_CH_3_)]I (3a).** Yield: 55%, yellow solid. ^1^H NMR (300 MHz, dmso-d_6_, 25 °C): δ = 7.97 (d, J = 3.6 Hz, 1H, H^6^), 7.42–7.32 (m, 2H, H^4,5^), 6.81 (t, J = 6.2 Hz, 1H, H^3^), 5.07 and 4.87 (AB system, J_AB_ = 16 Hz, 4H, N^+^CH_2_N), 4.69 (s, 2H, N^+^CH_2_N), 4.58–4.14 (m, 8H, NCH_2_N^+^NCH_2_P), 2.93 (m, 2H, N^+^CH_2_R), 1.7 (m, 2H, CH_2_), 1.30 (m, 10H, CH_2_), 0.86 (t, J = 6.8 Hz, 3H, CH_3_) ppm. ^31^P{^1^H} NMR (121.45 MHz, dmso-d_6_, 25 °C): δ = −36.8 ppm. δ = ^13^C{^1^H} NMR (75.4 MHz, dmso-d_6_, 25 °C): δ = 136.9, 129.1 and 116.5 (s, C_5_H_5_S), 80.1 and 79.1 (s, 2C, N^+^CH_2_N), 69.1 (d, J = 24 Hz, 1C, N^+^CH_2_P), 61.8 (s, 1C, N^+^CH_2_R), 48.1 (d, J = 10 Hz, 2C, NCH_2_P), 31.6 (s, 1C, C_intrachain_), 28.9 (s, 2C, C_intrachain_), 26.4 (s, 1C, C_intrachain_), 22.5 (s, 1C, C_intrachain_), 19.5 (s, 1C, N^+^CH_2_CH_2_R), 14.4 (s, 1C, CH_3_) ppm. Anal. calcd. (%) for C_19_H_33_AuIN_4_PS (704.40): C, 32.40; H, 4.72; N, 7.95; S, 4.40. Found: C, 23.58; H, 2.15; N, 5.71; S, 4.53.

**[Au(C_5_H_5_NS)(PTA-CH_2_CH_2_(CF_2_)_5_CF_3_)](TfO) (3b).** Yield: 50%, yellow solid. 1H NMR (400 MHz, dmso-d_6_, 25 °C): δ = 8.22 (d, J = 4.3 Hz, 1H, H^6^), 7.57 (m, 1H, H^4^), 7.49 (m, 1H, H^3^), 7.07 (t, J = 5.6 Hz, 1H, H^5^), 5.24–4.99 (m, 4H, NCH_2_N), 4.77–4.70 (m, 2H, N^+^CH_2_P), 4.58–4.54 (m, 2H, NCH_2_N), 4.39–4.13 (m, 4H, NCH_2_P), 3.0 (m, 2H, CH_2_-CF_2_). ^31^P{^1^H} NMR (162 MHz, dmso-d_6_, 25 °C): δ = −36.8 (s) ppm. ^13^C{^1^H} NMR (75.4 MHz, dmso-d_6_, 25 °C): δ = 167–110.4 (m series, 6C, -CH_2_-CF_2_-CF_2_-, -CH_2_-CF_2_-CF_2_-CF_2_-, -CH_2_-(CF_2_)_2_-CF_2_-, -CH_2_-(CF_2_)_3_-CF_2_-, -CH_2_-(CF_2_)_4_-CF_2_-CF_3_), 133.6, 127.8 and 119.3 (s, C_5_H_5_NS), 80.2 (s, 2C, N^+^CH_2_N), 68.9 (s, 1C, NCH_2_N), 49.5 (s, 1C, N^+^CH_2_R), 56.1 (d, J = 12.8 Hz, 1C, N^+^CH_2_P), 47.5 (d, J = 14.3Hz, 2C, NCH_2_P), 21.9 (t, J_CF_ = 20.7 Hz, 1C, N^+^CH_2_CH_2_R) ppm. ^19^F {^1^H} NMR (376 MHz, dmso-d_6_, 25 °C): δ = −77.8 (s, TfO^−^), −80.1 (m, CF_3_), −112.7 (m), −121.67 (s), −122.6 (m), −125.7 (m) ppm. Anal. calcd. (%) for C_20_H_20_AuF_16_N_4_O_3_PS_2_ (960.44): C, 25.01; H, 2.10; N, 5.83; S, 6.68. Found: C, 25.29; H, 1.94; N, 5.18; S, 6.74.

### 2.5. Distribution Coefficient (logD_7_._4_)

The n-octanol-water coefficients of the phosphane derivatives were determined as previously reported using a shake-flask method [34]. Briefly: Buffered-saline distilled water (100 mL, phosphate buffer [PO_4_^3−^] = 10 mM, [NaCl] = 0.15 M, pH 7.4) and n-octanol (100 mL) were shaken together for 72 h to allow saturation of both phases. 1 mg of the complexes was dissolved in 5 mL of the aqueous phase and 5 mL of the organic phase were added, mixing for 10 min. The concentration of the complexes in the organic and aqueous phases were then determined using UV absorbance spectroscopy. *LogD_7_._4_* = log{[compound(organic phase)]/[compound(aqueous phase)]}.

### 2.6. Solution Chemistry

The stability of the gold complexes has been analysed by absorption UV spectroscopy recorded on a Thermo Scientific spectrophotometer. Solutions of the new complexes (6 mM in DMSO) were diluted with PBS to led a final concentration of 3 × 10^−5^ M in PBS (pH = 7.4). The samples were then incubated at 37 °C and then monitored measuring the electronic spectra over 24 h.

### 2.7. Cell Culture, Cell Treatment and Cytotoxicity Determination

Human Caco-2 cell line (TC7 clone) was kindly provided by Dr. Edith Brot-Laroche (Université Pierre et Marie Curie-Paris 6, UMR S 872, Les Cordeliers, France). Human fibroblast cells were kindly provided by Dr. Julio Montoya (Unidad de Bioquímica, Facultad de Veterinaria, Universidad de Zaragoza, Spain). Caco-2 cells (passages 40–70) and fibroblasts (passages 10–30) were maintained in a humidified atmosphere of 5% CO_2_ at 37 °C. Cells were grown in Dulbecco’s Modified Eagles medium (DMEM) (Gibco Invitrogen, Paisley, UK) supplemented with 20% fetal bovine serum (FBS), 1% non-essential amino acids, 1% penicillin (1000 U/mL), 1% streptomycin (1000 μg/mL) and 1% amphotericin (250 U/mL). The cells were passaged enzymatically with 0.25% trypsin-1 mM EDTA. Caco-2 cells were sub-cultured on 25 cm^2^ plastic flasks at a density of 1.2 × 10^4^ cells/cm^2^, while fibroblasts were sub-cultured on 75 cm^2^ plastic flasks at a density of 1 × 10^4^ cells/cm^2^. Culture medium was replaced every 2 days.

Gold complex was diluted in dimethyl sulfoxide (DMSO) to a stock solution at 20 mM and then re-diluted to a working solution at 2.5 mM before diluting it in cell culture medium to treat cells at desired concentrations. A range of concentrations of complex 0.75–25 µM in Caco-2 and 1.25–20 µM in fibroblasts was used to determine IC_50_ values.

The influence of hyperthermia was studied by incubating cells at 40 °C for 1 h immediately after treating them with the gold complexes.

For cytotoxicity screening assays, Caco-2 cells were seeded in 96-well plates at a density of 4 × 10^3^ cells/well. Culture medium was replaced with medium containing drug panel 24 h post-seeding and cells were incubated for 72 h. In fibroblasts, culture medium containing metal gold complexes was added 10 days post-seeding. Antiproliferative effect was measured with MTT assay as previously described by Mármol et al. [16]. Absorbance at 540/620 nm was measured with SPECTROstar Nano (BMG Labtech). To determine the selectivity index (SI) the IC_50_ value in fibroblasts was divided by IC_50_ value in Caco-2, obtaining a ratio cancerous/normal cells toxicity.

### 2.8. Apoptosis Measurement

Caco-2 cells were seeded in 75 cm^2^ flasks at a density of 1 × 10^4^ cells/cm^2^ and then exposed to drug panel for 48 h, then collected and stained with Annexin V-FITC and propidium iodide according to manufacturer’s instruction. Cells were then transferred to flow cytometry tubes and washed twice with phosphate saline buffer (PBS), followed by a resuspension in 100 µL of annexing V binding buffer (100 mM Hepes/NaOH pH 7.4, 140 nM NaCl, 2.5 mM CaCl_2_). 5 µL annexin V-FITC and 5 µL propidium iodide (PI) were added to each tube. After 15 min of incubation at room temperature covered from light, 400 µL of annexin binding buffer were added to each sample and signal intensity was analysed within 1 h with BECKMAN COULTER GALLIOS (Brea, CA, USA). Data were analysed with BD FACSDivaTM.

### 2.9. Propidium Iodide Staining of DNA Content and Cell Cycle Analysis

Caco-2 cells were seeded in 25 cm^2^ flasks at a density of 1 × 10^4^ cells/cm^2^ and 24 h post-seeding, were exposed to drug panel for 48 h. Cells were fixed as described by Sánchez-de-Diego et al. [15] before staining them with 50 μg/mL PI solution. Signal intensity was analysed with a BECKMAN COULTER GALLIOS (Brea, CA, USA) equipped with a blue solid diode laser (488 nm) and a red solid diode laser (635 nm), collecting fluorescence with a 620 nm long pass filter. Cell distribution was displayed on a linear scale and analysed with ModFit LTTM verity software, which determined the percentage of cells in every cycle phase.

### 2.10. Mitochondrial Membrane Potential Assay by Flow Cytometry

Caco-2 cells were seeded in 75 cm^2^ flasks at a density of 1 × 10^4^ cells/cm^2^ and 24 h post-seeding medium was replaced with medium containing drug panel and incubated for 48 h. Then. cells were washed twice and resuspended in PBS at a concentration of 10^6^ cells/mL before incubation with 5 µL of 10 µM 1,1′,3,3,3′-hexamethylindodicarbo-cyanine iodide (DilC1). After 15 min incubation at 37 °C, 400 µL were added and fluorescence was measured at excitation wavelength of 633 nm and emission of 658 nm with BECKMAN COULTER GALLIOS (Brea, CA, USA) equipped with a blue solid diode laser (488 nm) and a red solid diode laser (635 nm).

### 2.11. Determination of Caspase 3 and p53 Proteins

Caco-2 cells were seeded in 75 cm^2^ flask at a density of 1 × 10^4^ cells/cm^2^ and, 24 h later, exposed to drug panel for 48 h. Then cells were collected and processed following instructions by Sánchez-de-Diego et al. [15] Finally, 50 µL of every sample were incubated with 5 µL anti-active caspase-3 (BD Pharmigen, Clone C92-605) and 5 µL p53 antibody (Miltenyi, Clone REA609). Fluorescence was measured by flow cytometry using a BECKMAN COULTER GALLIOS (Brea, CA, USA) equipped with a blue solid diode laser (488 nm) and a red solid diode laser (635 nm). For caspase-3 determination excitation wavelength was set at 488 nm and emission at 525 nm and for p53 analysis excitation at 635 nm and emission at 660 nm.

### 2.12. Intracellular Levels of Reactive Oxygen Species (ROS)

Caco-2 cells were seeded in 96-wells plate at a density of 4 × 10^3^ cells/well and intracellular ROS levels were determined with the dichlorofluorescein assay [35]. Cells were exposed to drug panel for 1, 3 and 24 h and then incubated with 20 µM 2′-7′-dichlorofluorescein diacetate (DCFH-DA) (Merck KGaA, Darmstadt, Germany) in DMEM. The generation of oxidised derivative DCF was monitored by measuring the increase of fluorescence for 1 h, at an emission wavelength of 520 nm and excitation of 485 nm, with a FLUOstar Omega (BMG Labtech, Ortenberg, Germany) multiplate reader. Results were expressed as percentage of fluorescence respect to control, considering fluorescence intensity as a reflection of intracellular ROS levels.

### 2.13. Thioredoxin Reductase 1 (TrxR1) Activity Assay

Recombinant Human TrxR1 (Sigma-Aldrich SRP6081, St. Louis, MO, USA) was incubated in a 96 well plate with different concentration of Au complex previously dissolved in 25 μL of PBS at pH 7.4. The cell solution was incubated in shake motion for 5 min at 37 °C. After incubation time, procedure in Thioredoxin Reductase Assay Kit (Sigma-Aldrich CS0170, St. Louis, MO, USA) was followed. Finally, reaction was started by adding 6 μL DNTB and absorbance at 405 nm was recorded every 30 s for 22 min as a measure of Thioredoxin Reductase Activity.

### 2.14. Statistical Analysis

All assays were performed at least three times. Data are presented as mean ± SD. Means were compared using one-way analysis of variance (ANOVA). Significant differences at *p* < 0.05 were compared using a Bonferroni’s Multiple Comparison Test. The statistical analysis and the graphics were performed using the GraphPad Prism Version 5.02 program on a PC computer.

## 3. Results and Discussion

### 3.1. Synthesis of the PTA Molecules

The N-alkyl PTA molecule [PTA-CH_2_(CH_2_)_6_CH_3_]I (**La**) was synthesised by reacting 1,3,5-triaza-7-phosphaadamantane (PTA) with 1-iodooctane in acetone degassed under argon for a long period of time. The initial use of heat, in order to improve the reaction rate, lead to PTA decomposition. After stirring for 3 days at room temperature, an air-stable solid precipitated from solution, which was isolated and characterised without further purification. Similar [PTA-R]I phosphanes with longer chains (R = C_n_H_2n+1_, n = 12, 16, 18) have been previously synthesised [36] under continuous bubbling of argon for 20 h. The occurrence of the reaction was monitored by means of ^1^H NMR and ^31^P{^1^H} NMR. In the ^31^P{^1^H} NMR spectrum a singlet shifted downfield from −102 ppm in the original PTA to around −85 ppm (alkylated PTA) was observed. The ^1^H NMR shows a typical pattern for N-alkylated PTA molecules [34,36,37,38,39], due to the splitting of different signals consequence of the reduction of the symmetry in the PTA molecule and the appearance of the signals related to the aliphatic chain (see experimental).

Any attempt to N-alkylation with the corresponding perfluorinated chain, by reacting PTA with 1H,1H,2H,2H-Perfluoro-1-iodooctane, turned out to be infructuous, leading to PTA oxidation. However, the use of more reactive alkyl triflates, such as TfOCH_2_CH_2_(CF_2_)_5_CF_3_, which undergo facile displacement of the triflate anion via C-O bond cleavage [40], afforded [PTA-CH_2_CH_2_(CF_2_)_5_CF_3_]TfO (**Lb**) in high yield. Similar NMR spectra to that found for La were obtained for Lb, although only the signals due to methylene groups –CH_2_ α (3.20 ppm) and –CH_2_ β (2.93 ppm) to quaternary nitrogen atom, are visible in the ^1^H NMR spectrum. The IR spectrum gave additional evidence for the N-perfluorinated alkylation showing bands at 1224 and 1142 cm^−1^, corresponding respectively to C-F bonds symmetric and asymmetric stretching frequencies. Besides, ^19^F{^1^H} NMR showed six signals, due to the –(CF_2_)_5_CF_3_ chain and ^13^C{^1^H} NMR multiplets in the region of 121.3–108.2 ppm that correspond to 6C of the perfluorinated chain.

### 3.2. Synthesis of Gold(I) Derivatives

**Scheme 1:**
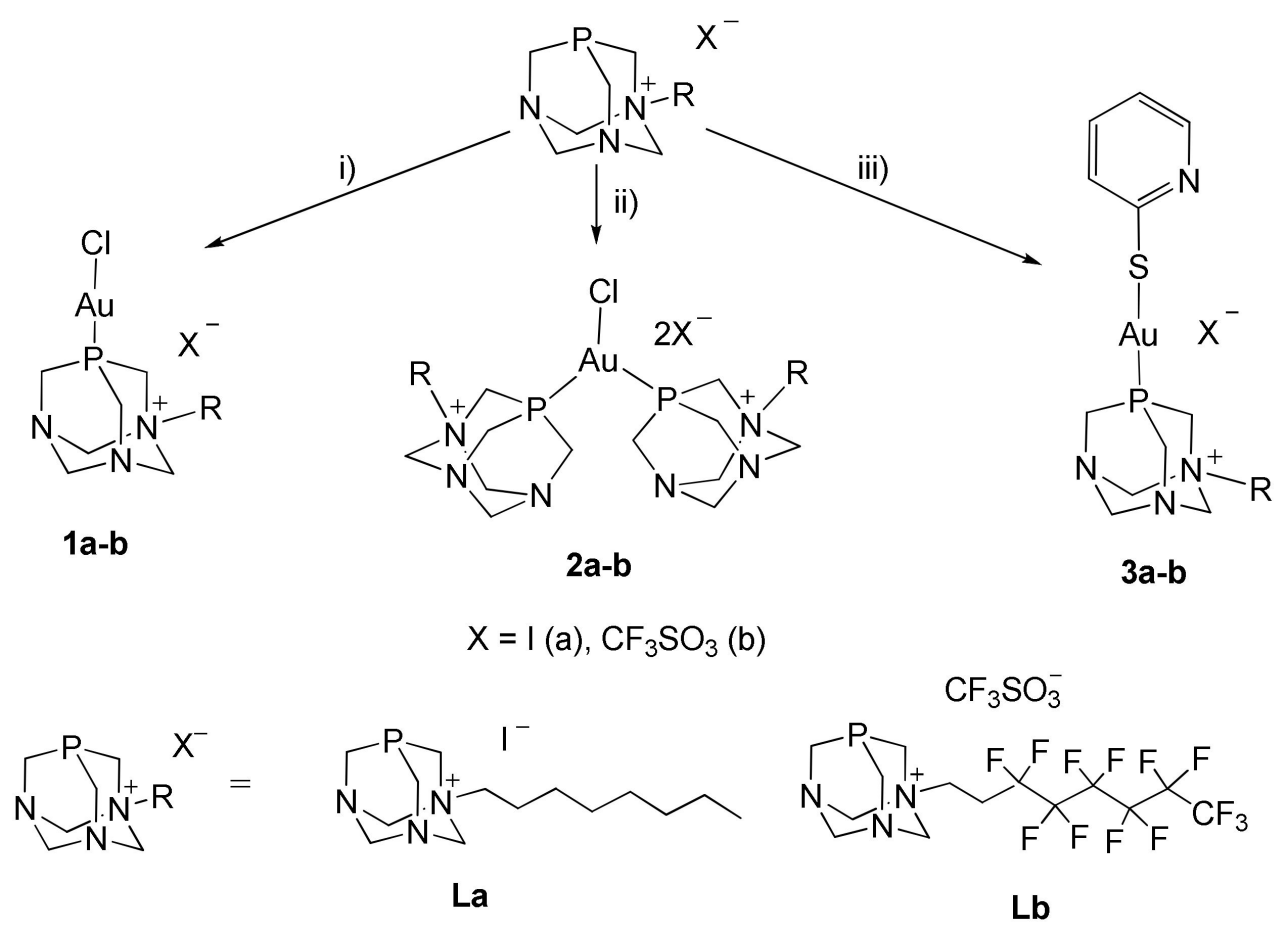
Synthesis of gold complexes. (i) [AuCl(tht)], (ii) [AuCl(PTA-R)]X, (iii) HSC_5_NH_4_ + NaOEt + [AuCl(tht)], then [PTA-R]X.

Coordination of the phosphanes to gold(I) is achieved by the reaction of **La** and **Lb** with [AuCl(tht)] (tht = tetrahydrothiophene) (Scheme 1, (i)) after the displacement. The ^1^H NMR spectra are markedly affected by the presence of the Au-Cl unit, similarly to related chloro gold(I) derivatives with alkylated PTA molecules [34,38]. Thus, the AB system corresponding to the methylene NCH_2_N^+^ protons remains at a similar chemical shift, meanwhile the remaining protons appear as multiplets due to the overlap of doublets, displaced to low field in comparison to those of the free phosphanes. Downfield displacement is also detected in their ^31^P{^1^H} NMR spectra in the relation to the corresponding free phosphane, which is more pronounced in 1a, with a singlet resonance at δ = –30 ppm, being δ = –35.8 ppm for the related perfluorinated counterpart **1b**. The Au-Cl vibrations at around 333–320 cm^−1^ are visible in their IR spectra and the corresponding molecular peaks are observed in the MALDI^+^ mass spectra, without the presence of additional peaks due to species obtained from an interchange between both anions.

The reaction of **La** or **Lb** with the corresponding chloro gold complexes **1a** and **1b** afforded [AuCl(PTA–R_2_)]X_2_ (**2a**, **2b**, Scheme 1, (ii)), where two phosphanes are coordinated to the Au-Cl unit in a trigonal disposition. Similar pattern than that found in the precursors is observed in their ^1^H NMR spectra, with even higher signals overlapping. The ^31^P{^1^H} NMR spectra display upfield displacement of the resonances with respect to the chloro gold precursors. The Au-Cl vibration at 320 cm^−1^ is only observed in the IR spectrum of complex **2b**, being the same band absent in complex **2a**. This fact could point to the existence of a fast anion interchange between chlorine and iodine in solution, affording the isolation of [AuI(PTA-R)_2_]ClI. This occurrence can be confirmed with the MALDI^+^ experiment, since the presence of the peak with *m*/*z* of 594.7 can be assigned to the loss of [PTA-R]I and chlorine units.

The synthesis of the thiolate complexes **3a** and **3b** was performed as previously reported by some of us [13]. So, starting from the insoluble oligomeric or polymeric [{Au(thiolate)}n], prepared in situ by deprotonation of pyridine-2-thiol and subsequent addition of [AuCl(tht)], followed by the addition of the phosphanes once the subproducts were removed by decantation of the supernatant, afforded the new thiolate derivatives [Au(PTA-R)(SNC_5_H_4_)]X (X = I, **3a**; X = TfO, **3b**) (Scheme 1, (iii)), which were fully characterised by NMR, IR spectroscopy.

### 3.3. Solution Stability

The stability of the complexes was analysed by UV-vis absorption spectroscopy under physiological relevant conditions. Solutions suitable for spectrophotometric analyses were prepared by diluting dimethylsulfoxide (DMSO) stock solutions of the complexes with PBS (phosphate buffered saline) at pH = 7.4. The resulting solutions were monitored over 24 h at 37 °C. The UV-visible absorption spectra of the complexes show an absorption maximum at ca. 210–220 nm (Figure S39), which can be tentatively assigned to the intraligand transition characteristic of the phosphane, as similar absorption bands are also observed in related Au(PTA) complexes [18]. For all the complexes no changes were observed in the UV-vis absorption spectra with time, without any changes in shape or displacement in the absorbance maximum (no apparent red- or blue shift), in addition to lacking of absorbance at around 500 nm (characteristic of gold reduction), thus indicating a substantial stability in the selected conditions.

### 3.4. Lipophilicity

These new derivatives are mono or dicationic species, and subsequently they belong to the class of delocalised lipophilic cations (DLCs) [41], characterised by a delocalised positive charge, lipophilic character and rigid structure. These molecules display rapid accumulation in mitochondria leading to alterations in their functions, in response to the negative charge inside the transmembrane potentials [42,43]. In addition, tuning the lipophilicity and hydrophilicity character of the DLC moieties, can selectively target cancer cells due to their higher mitochondrial membrane potential compared to normal cells [44]. Previous studies based on cationic gold(I) derivatives have revealed the relevance of the lipophilicity in the cellular uptake and cytotoxicity, and how intermediate values in the lipophilic character lead to more promising conditions to avoid severe side-effects [7,45,46].

The lipophilicity can be measured by the partition coefficient water/n-octanol, *logD,* by experimental procedures such as the shake flask method, which affords the distribution of the drug between an equal amount of n-octanol and water (or buffered aqueous solution). The new PTA derivatives display values of *logD* (at pH = 7.4) in the range −0.12 to 0.82 (Table 1), being higher in the cases of the polyfluorinated phosphanes that implies higher lipophilicity in comparison with the alkylated counterparts.

### 3.5. Biological Studies

The potential anticancer properties of these new gold(I) derivatives were analysed using human carcinoma cell line Caco-2. The cytotoxic effect of the gold complexes on these cells was evaluated by determining IC_50_ values (necessary concentration to reduce two-fold cell viability) (Table 1) and then analysing induced cell death and potential cellular target.

#### 3.5.1. Antiproliferative Activity of Gold(I) Derivatives with PTA

The results showed that our compounds exhibit an IC_50_ in the range of other gold complexes previously studied [47,48,49], thus they have potential antiproliferative properties. Although every tested compound suffered a decrease in the IC_50_ value when cells were incubated at 40 °C for 1 h, this decrease was especially significant for **2a** and **2b**. However, the presence of the perfluorinated chain in the complexes entailed significance differences in the IC_50_ values compared to the alkylated counterparts. IC_50_ decreased in **Lb**, **1b** and **2b**, but increased in **3b** (Table 1).

A correlation between lipophilicity and cytotoxicity was found, obtaining higher IC_50_ values in compounds **1a** and **2a**, which display the lower *logD* values. Higher lipophilicity correlates with more cytotoxic compounds and species **3a** and **3b**, with the lower IC_50_ values, showed intermediate lipophilic character (Table 1). When increasing lipophilic character, with increasing *logD* values, the activities of the complexes describe an inverted U-shaped curve (Figure S40, Supplementary). Accordingly, the highest anticancer activity should be expected for complexes with intermediate *logD* values, around 0.4.

Complexes **3a** and **3b** triggered the best activity, exhibiting the lowest IC_50_ values despite being the only couple of complexes that did not show a lower value for the perfluorinated chain complex and only a slight decrease in the IC_50_ values in hyperthermia conditions. Contrary, complexes **2a** and **2b** showed worse IC_50_ values, but the trend follows as expected a decrease in the activity of **2b** in comparison to **2a**, since **Lb** showed higher toxicity than **La** and a significant reduction of IC_50_ values with hyperthermia. To elucidate the relationship in the activity of complex pairs in deep, the cytotoxic activity in relation to selectivity of **2a**, **2b**, **3a** and **3b**, was analysed.

IC_50_ values in fibroblasts showed that complexes **3a** (12.11 ± 0.11 µM) and **3b** (12.82 ± 1.48 µM) had a selectivity index (SI) of 4.86 and 2.51, respectively, thus being more selective towards cancerous cells than complexes **2a** (13.47 ± 0.97 µM) and **2b** (11.59 ± 1.05 µM), whose SI were 1.09 and 1.72, respectively (Table 2). Moreover, at their IC_50_ values in Caco-2, **3a** (2.5 µM) and **3b** (5.1 µM) did not compromise fibroblasts viability. On the contrary, complexes **2a** and **2b** caused a nearly two-fold reduction in the viability of fibroblasts when treated at 12.3 µM and 10.5 µM, respectively. These results suggest that, unlike **2a** and **2b**, **3a** and **3b** could selectively exert their cytotoxic effect in cancerous cells without causing several side effects in normal cells. As observed in Table 2, IC_50_ values on fibroblasts are very similar for every tested compound. This fact suggests that the differences among tested compounds do not have a marked influence on the cellular uptake, or the interaction with intracellular targets and consequent influence on cell proliferation. However, **3a** and **3b** have lower IC_50_ values on Caco-2 cells, thus, it is in cancerous cells where we find the difference in cytotoxic action. One feasible explanation for this is, as previously proposed, the lipophilicity, which falls in mid-levels for **3a** and **3b**, whereas *logD_7.4_* values for **2a** and **2b** are either lower (**2a**) or higher (**2b**).

Taking into consideration these analyses, we chose complexes **3a** and **3b** to dig into the study of the influence of temperature and perfluorinated chains in the cytotoxic and anticancer properties of these gold compounds.

#### 3.5.2. Cell Death Induced by Gold(I) Derivatives with PTA

Since the complexes compromised cell proliferation, different flow cytometry determinations were performed in order to assess the type of cell death that these gold compounds induced in Caco-2 cells, as well as confirm the previously suggested influence of temperature and fluorinated chains in the cytotoxic capacity of the complexes.

**Figure 1:**
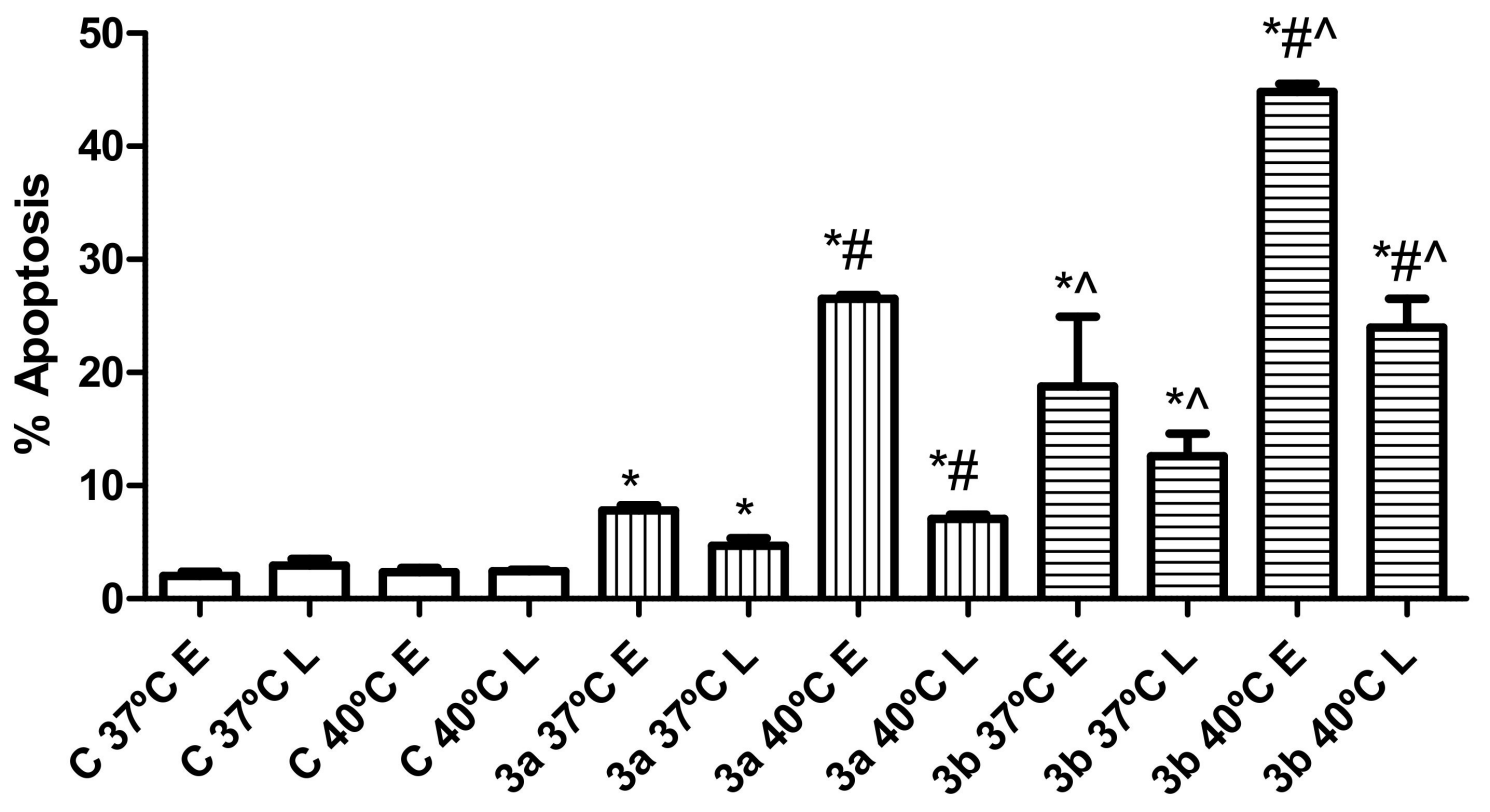
Caco-2 cell death studies by flow cytometry after incubation with **3a** (5.0 µM) and **3b** (10.2 µM) for 48 h at 37 °C or 1 h at 40 °C plus 47 h at 37 °C. Results of apoptotic state are expressed as percentage of cells going through early (E) and late (L) apoptosis for each condition. * *p* < 0.05 compared to respective temperature control. # *p* < 0.05 compared to equivalent treatment at 37 °C. ^ *p* < 0.05 compared to alkylated counterpart at same temperature.

First, Annexin V/PI double staining showed that gold compounds were capable of inducing apoptosis in Caco-2 cells after 48 h of treatment, with prevalence of cells in early apoptotic stages (Figure 1). Moreover, statistical analysis of the results showed that 1 h of incubation at 40 °C immediately after exposure to gold complexes, significantly increased the amount of cells in early and late apoptosis, and this effect was also observed in complex **3b** compared to **3a** (Figure 1).

**Figure 2:**
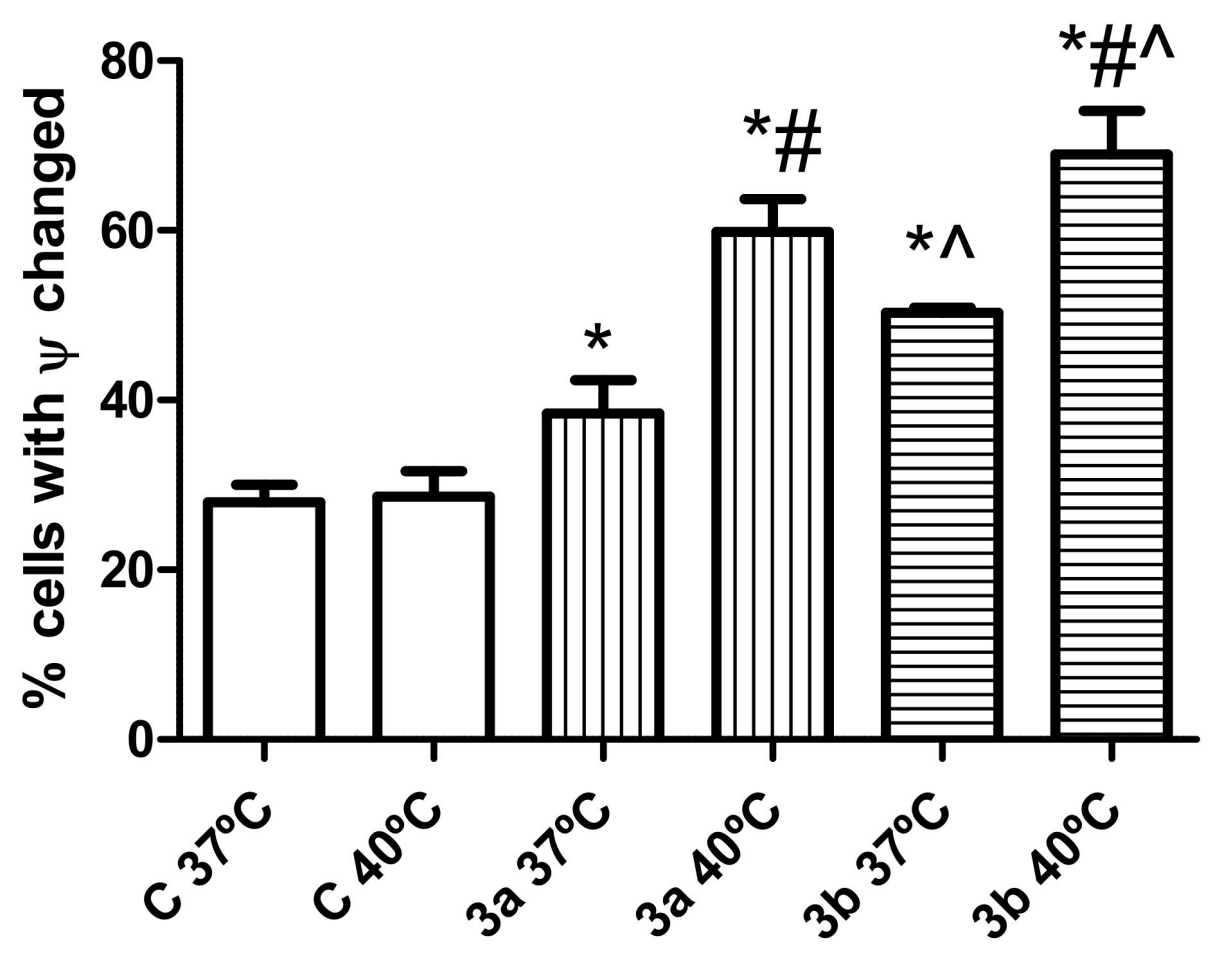
Percentage of Caco-2 cells with disturbed mitochondrial potential (Ψ) after incubation with **3a** (5.0 µM) and **3b** (10.2 µM) for 48 h at 37 °C or 1 h at 40 °C plus 47 h at 37 °C. * *p* < 0.05 compared to respective temperature control. # *p* < 0.05 compared to equivalent treatment at 37 °C. ^ *p* < 0.05 compared to alkylated counterpart at same temperature.

One of the first steps that leads to apoptosis is mitochondrial depolarisation. Throughout the apoptotic process the mitochondrion undergoes the redistribution of hydrogen ions, reducing ΔΨm, which induce series of structural changes that finally lead to cytochrome c release. Results from DilC1(5) staining showed that both complexes **3a** and **3b** induced mitochondrion depolarisation, since the number of Caco-2 cells with reduced ΔΨm increased after 48 h exposure to both gold compounds (Figure 2). In addition, statistical analysis brought significant differences between hyperthermia and normothermia, as well as with perfluorinated and alkylated complexes (Figure 2). This confirmed results from Figure 1, suggesting that complex **3b** exert a higher cytotoxic effect than **3a** and that 1 h incubation with complexes at 40 °C increase the apoptotic effect, regardless of the presence of perfluorinated or alkylated chains in gold compounds.

**Figure 3:**
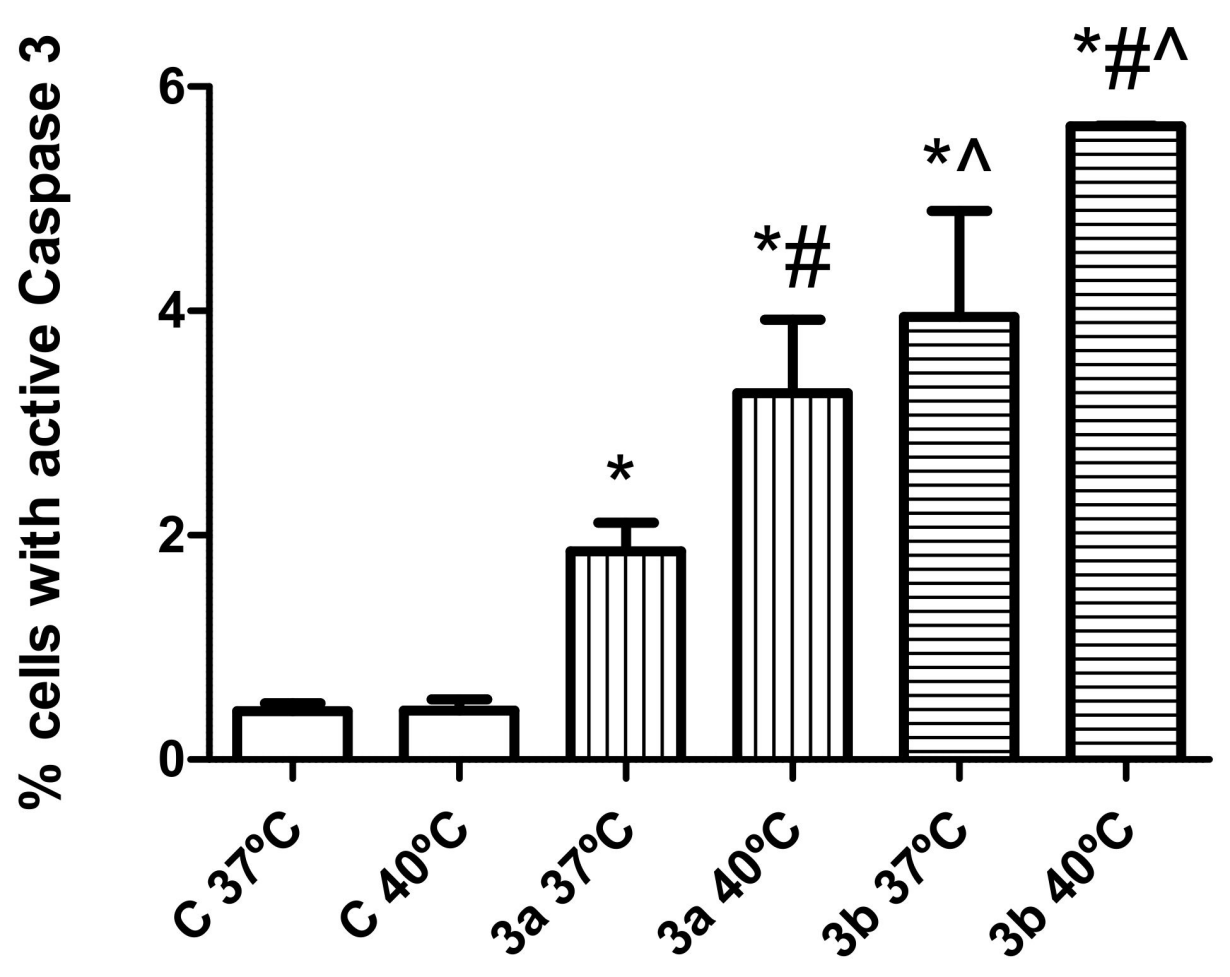
Percentage of Caco-2 cells with presence of active caspase-3 after incubation with **3a** (5.0 µM) and **3b** (10.2 µM) for 48 h at 37 °C or 1 h at 40 °C plus 47 h at 37 °C. * *p* < 0.05 compared to respective temperature control. # *p* < 0.05 compared to equivalent treatment at 37 °C. ^ *p* < 0.05 compared to alkylated counterpart at same temperature.

Mitochondrial depolarisation and cytochrome c release finally leads to caspase-3 activation, which starts the apoptotic process. Flow cytometry determinations confirmed previous results, since incubation with both gold complexes for 48 h increased the percentage of cells with active caspase-3 (Figure 3). This suggests that **3a** and **3b** could induce caspase-3 activation upon ΔΨm reduction, finally leading to apoptosis in Caco-2 cells. Moreover, significant changes were found again with hyperthermia treated cells and with the perfluorinated-chain compound treated cells.

#### 3.5.3. Effect of Gold(I) Derivatives with PTA on Cell Cycle

Apoptosis events frequently are accompanied by cell cycle disturbances and previous studies with gold complexes suggested that treated cells may stop progression through cell cycle phases [18,50,51] at different checkpoints along them.

**Figure 4:**
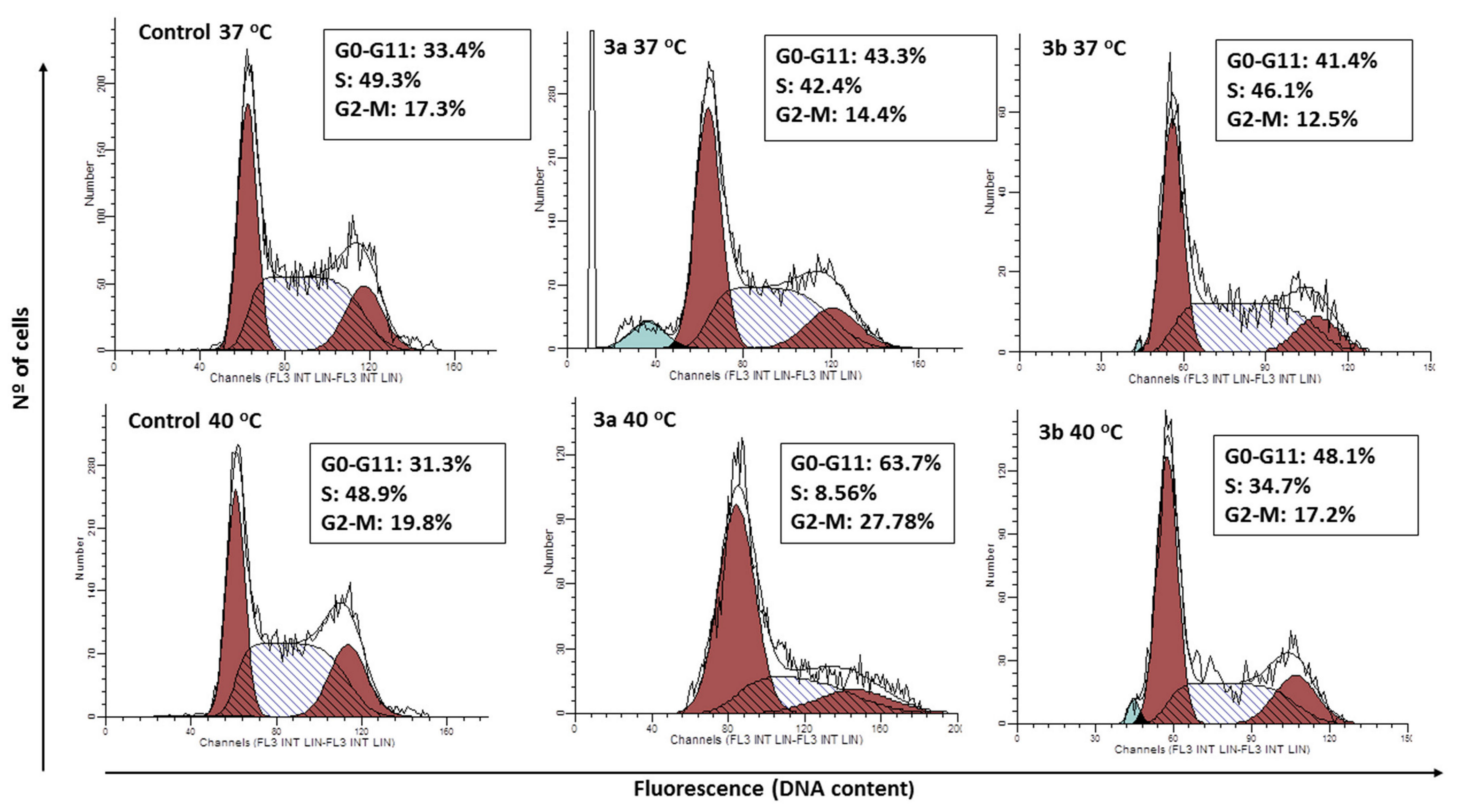
Flow cytometry histograms of PI stained Caco-2 cells after 48 h treatment with **3a** (5.0 µM) and **3b** (10.2 µM) at 37 °C or 1 h at 40 °C plus 47 h at 37 °C. Distribution of cells along cell cycle phases.

Analysis by flow cytometry showed that after 48 h incubation with complexes **3a** and **3b** could disturb normal progression through cell cycle, increasing amount of cells in G0-G1 phase (Figure 4). In addition, hyperthermia and fluorinated chains seemed to enhance G1 phase arrest.

**Figure 5:**
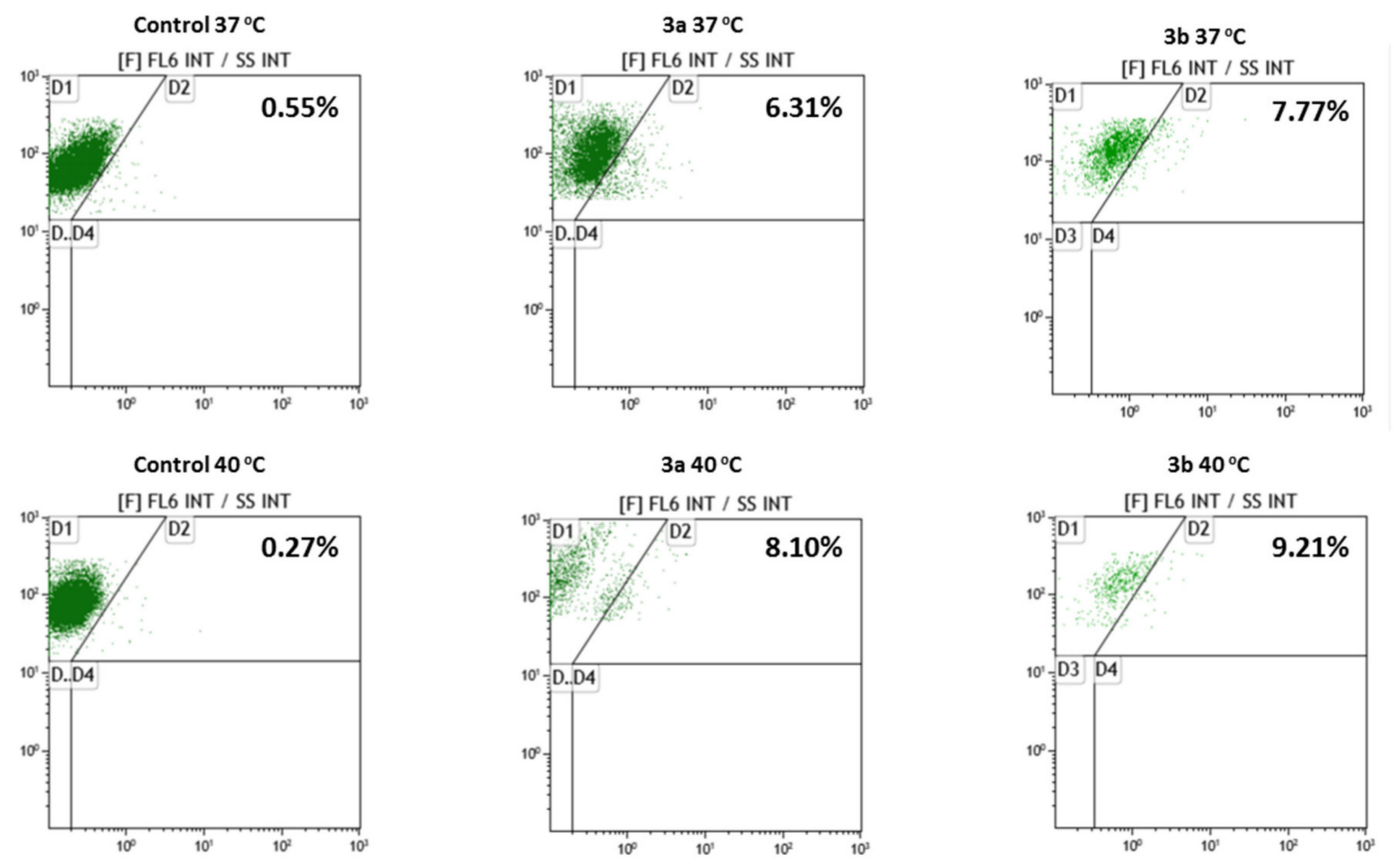
Flow cytometry cytograms of anti-p53 fluorescence in Caco-2 cells after exposure to **3a** (5.0 µM) and **3b** (10.2 µM) for 48 h at 37 °C or for 1 h at 40 °C plus 47 h at 37 °C. Quadrant D2 correspond to cells with active p53, which percentage respect to total cells is represented.

Along the cell cycle, cells go through different checkpoints that ensure order, integrity, and fidelity in the previous events before progress through cell cycle and promoting to the next phase [52]. One of the main checkpoint mechanisms in G1 phase is led by p53, a transcription factor that targets transcription of genes involved in G1 arrest. Levels of p53 are regulated by MDM2, an ubiquitin ligase that ensures p53 proteasome-dependent turnover. When genotoxic stress is detected, this checkpoint response is started, activating upstream events that lead MDM2 activation thus promoting p53 accumulation and sustained G1 arrest [53]. Moreover, p53 can induce apoptosis cell death in response to DNA damage [54]. Taking all this in consideration, the presence of p53 in cells treated with complexes **3a** and **3b** was determined, observing that these gold derivatives could induce p53 accumulation, increasing the number of cells with p53 with respect to non-treated cells (Figure 5). These results correlate with previous showed in Figure 4 and incubation with gold derivatives at hyperthermia increased p53 in cells, as well as polyfluorinated chains did compared to the alkylated counterpart (Figure 5).

Taking all these results into consideration, hyperthermia and fluorinated chains seem to have an impact in cytotoxicity. We suggest that hyperthermia can influence on cell function and shape at different levels, enhancing drug uptake and accumulation inside cells, as previous studies have proved. Moreover, hyperthermia could also affect cell organelles (mitochondria, nucleus) and potential intracellular targets making them more sensitive to our gold compounds. [55,56,57] With regard to fluorinated chains, fluor is proposed to stabilize the molecule, then increasing its biological activity once in the cell. [58]

#### 3.5.4. Effect of Gold(I) Derivatives with PTA on Intracellular Redox State

**Figure 6:**
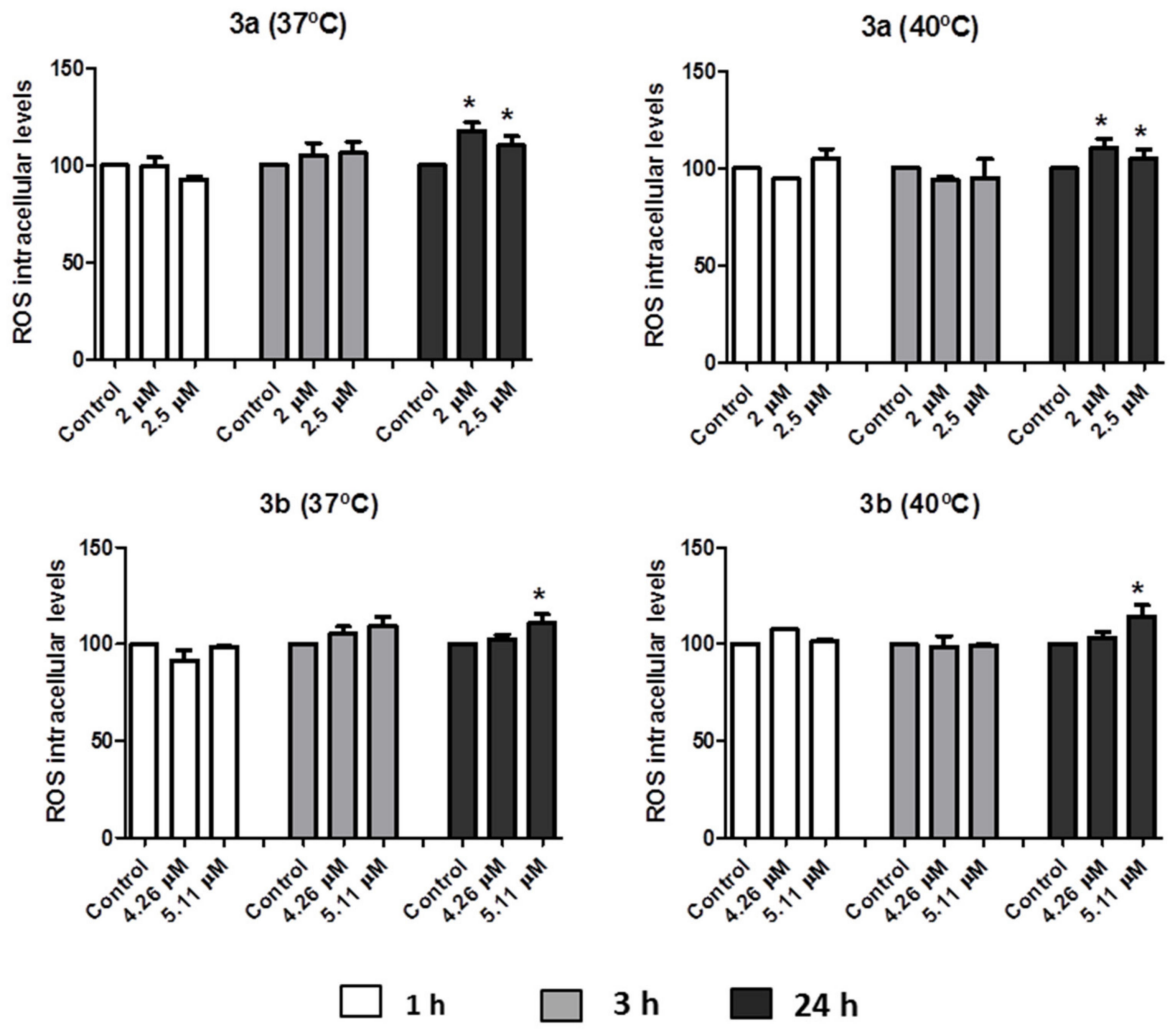
Measurements of ROS levels in Caco-2 cells after expsoure to **3a** and **3b** complexes for 1, 3 or 24 h at 37 °C or hyperthermia, * *p* < 0.05 compared to its respective control.

Previous studies with gold derivatives had shown disturbances in ROS balance when cancerous cells were treated with metal complexes [16,55,56], commonly increasing intracellular ROS levels, since different gold(I) derivatives have been proved to interact with thioredoxin reductase (TrxR) inhibiting its antioxidant activity [14,56]. Our results did not bring significant changes in intracellular ROS levels when cells were treated with complexes **3a** and **3b** for 1 h and 3 h, but a significant increase was observed when treated for 24 h with IC_50_ values. However, significant differences between ROS levels at 2 µM and 2.5 µM were not found for **3a** complex, but concentration seemed to be important for complex **3b** (Figure 6). Concentrations assayed correspond to IC_50_ values at 37 ºC and 40 ºC, instead of two-fold IC_50_ values used for cytometry assays, because ROS-levels-increase is previous to apoptosis and cytotoxic concentrations are not desired.

**Figure 7:**
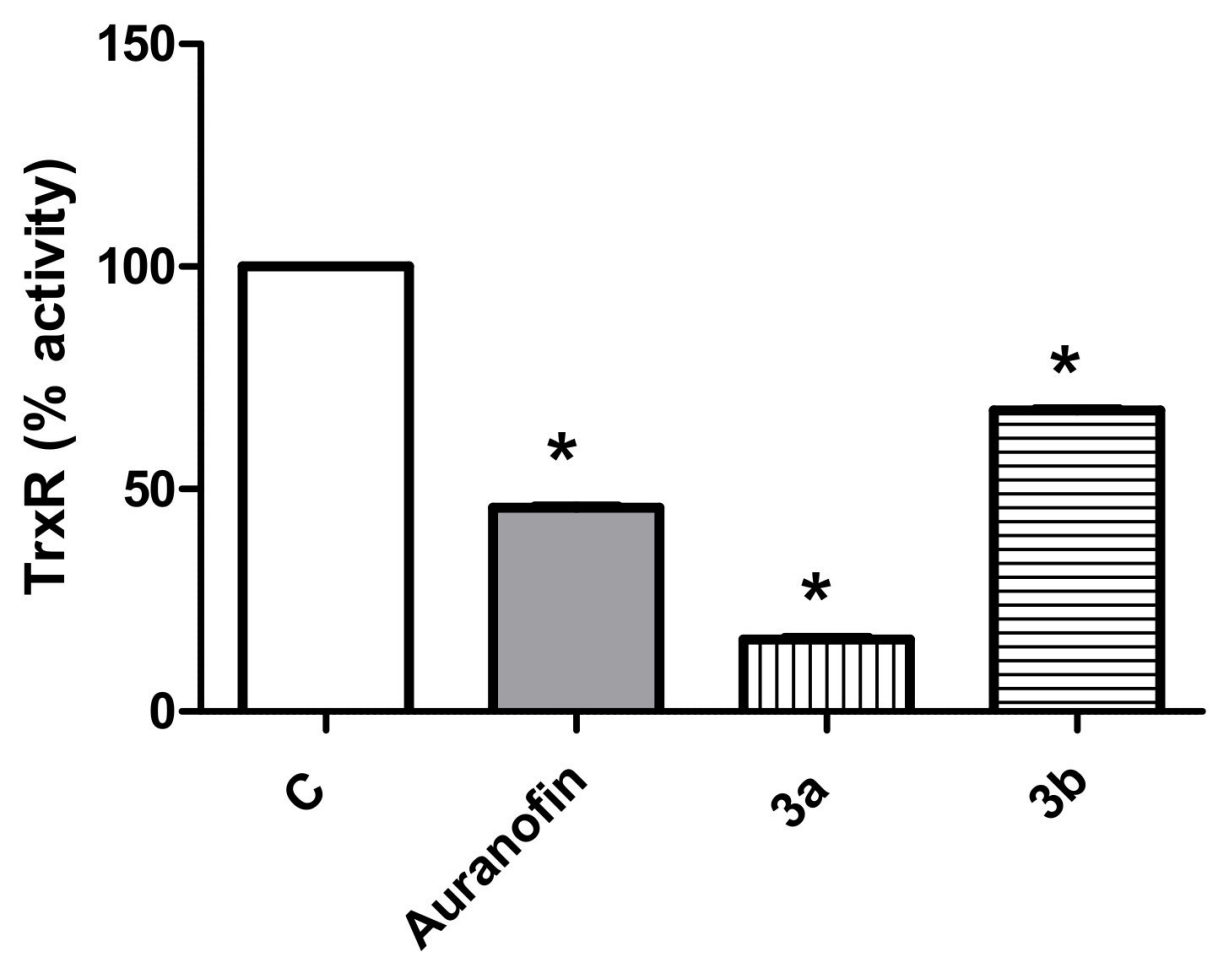
Inhibitory effect of auranofin (25 µM), **3a** and **3b** complexes on Thioredoxin reductase 1 (TrxR1) at IC_50_ concentrations. TrxR activity was measured as the increase of absorbance at 412 nm per minute. Values represent the percentage of activity related to control, * *p* < 0.05 compared to control.

To elucidate whether this ROS increase could be caused by TrxR, recombinant human TrxR was incubated with both derivatives at their respective IC_50_ values, detecting an inhibition in TrxR activity (Figure 7). Therefore, our results suggest that these gold(I) derivatives could interact with TrxR, inhibiting its antioxidant activity, hence increasing intracellular ROS levels.

## 4. Conclusions

We have synthesized and fully characterized six new gold(I) derivatives and two new alkylated PTA (1,3,5-triaza-7-phosphaadamantane) ligands functionalised with long hydrocarbon chains and with the homologous fluorinated chains with the purpose of knowing the influence of the fluorine atoms and an increase of the temperature, hyperthermia, in the activity. All of them, including the free ligands, have been tested against human carcinoma cell line Caco-2. The studied gold(I) complexes showed antiproliferative effects on human colon carcinoma cells (Caco-2/TC7 cell line), probably by inhibiting cellular TrxR activity, causing a dysfunction in the intracellular redox state. This event produces disturbances in cell cycle by p53 activation. Finally, apoptosis is triggered through mitochondrial depolarisation and the consequent activation of caspase-3. Moreover, the results suggest that this cytotoxic effect is enhanced by hyperthermia, obtaining lower IC_50_ values and higher cell death rates when incubating cells for 1 h at 40 °C after the addition of the drug. The presence of perfluorinated chains appeared also to be important since gold(I) complexes with fluorinated chains brought higher cytotoxic results than their alkylated counterparts.

This work is defined in a larger project about treatment and prevention of colorectal cancer, comparing inorganic compounds to natural compounds from vegetal extracts. It would be interesting to assess the cytotoxic activity of these gold derivatives in different cancerous cell lines, but at present our framework is within colon cancer.

## Figures and Tables

**Table 1:** Distribution coefficients and IC_50_ (M) ^[a]^ values of the complexes on Caco-2/TC7 cells.

Complex	*logD_7.4_*	Caco-2/TC7	
		37 °C	40 °C
**La**		49.32 ± 0.02	47.08 ± 0.92
**Lb**		27.23 ± 7.56	23.97 ± 6.60
**1a**	0.08	16.41 ± 3.11	13.04 ± 4.24
**1b**	0.63	5.98 ± 1.80	4.46 ± 0.79
**2a**	−0.12	12.34 ± 1.18	10.39 ± 0.51
**2b**	0.82	10.65 ± 0.60	9.00 ± 0.39
**3a**	0.35	2.49 ± 0.31	2.05 ± 0.32
**3b**	0.44	5.11 ± 1.25	4.26 ± 0.34

^[a]^ Mean ± SE of at least three determinations.

**Table 2:** Selectivity of complexes **2a**, **2b**, **3a**, and **3b** towards cancerous cells. IC_50_ (M) ^[a]^ values on fibroblasts are presented. Selectivity Index (SI) was calculated as IC_50_ fibroblasts/IC_50_ Caco-2.

Complex	Fibroblasts	
	IC_50_	SI
**2a**	13.47 ± 0.97	1.09
**2b**	11.59 ± 1.05	1.72
**3a**	12.11 ± 0.11	4.86
**3b**	12.82 ± 1.48	2.51

^[a]^ Mean ± SE of at least three determinations.

## Data Availability

Data is contained within the article or supplementary material.

## Supplementary Materials

The following are available online at www.mdpi.com/article/10.3390/biomedicines9121848/s1, Figures S1–S38: ^1^H, ^31^P{^1^H}, ^13^C{^1^H} and HSQC NMR spectra of gold(I) complexes, Figure S39: UV-Vis spectra from stability assays, Figure S40: Correlation between cytotoxic activity of the complexes and logD_7_._4_.
