# Supplementary material for: Gold(I) Complexes Bearing Alkylated 1,3,5-Triaza-7-phosphaadamantane Ligands as Thermoresponsive Anticancer Agents in Human Colon Cells
Source: Biomedicines. 2021 Dec 6;9(12):1848. doi: 10.3390/biomedicines9121848 (PMC8698759; doi:10.3390/biomedicines9121848)
Supplement: Supplementary file 1 [file biomedicines-09-01848-s001.zip › biomedicines-1470706-supplementary.pdf]

Gold(I) complexes bearing alkylated 1,3,5-triaza-7-phosphaadamantane ligands as thermoresponsive anticancer agents in colon cells

Javier Quero,[1,2] Francesco Ruighi, [1] Jesús Osada, [3] M. Concepción Gimeno,[1] Elena Cerrada,[1]*and Maria Jesús Rodriguez-Yoldi,[2]*

[1] Departamento de Química Inorgánica, Instituto de Síntesis Química y Catálisis Homogénea-ISQCH, Universidad de Zaragoza-C.S.I.C., 50009 Zaragoza, Spain. E-mail: ecerrada@unizar.es ; francesco.ruighi@edu.unito.it and gimeno@unizar.es

[2] Departamento de Farmacología y Fisiología y Medicina Legal y Forense. Unidad de Fisiología, Universidad de Zaragoza, 50013, Zaragoza, Spain, CIBERobn, IIS Aragón, IA2. E-mail: mjrodyol@unizar.es and javierquero94@gmail.com

3 Departamento de Bioquímica y Biología Molecular. Unidad de Bioquímica. Universidad de Zaragoza, 50013, Zaragoza, Spain, CIBERobn, IIS Aragón, IA2. E-mail: josadal@unizar.es

* Correspondence: mjrodyol@unizar.es and ecerrada@unizar.es

Citation: Quero, J.; Ruighi, F.; Gimeno, M.C.; Cerrada, E., Rodriguez-Yoldi, M.J.Thermoresponsive gold derivatives as anticancer agents. *Biomedicines* **2021**, *9*, x. https://doi.org/10.3390/xxxxx

Academic Editor: Firstname Lastname

Received: date
Accepted: date
Published: date

Publisher's Note: MDPI stays neutral with regard to jurisdictional claims in published maps and institutional affiliations.

Copyright: © 2021 by the authors. Submitted for possible open access publication under the terms and conditions of the Creative Commons Attribution (CC BY) license (https://creativecommons.org/licenses/by/4.0/).

Table of Contents

Figures S1-S38.^{1}H, ^{19}F{^{1}H}, ^{31}P{^{1}H}, ^{13}C{^{1}H} and HSQC NMR spectra of gold(I) complexes

Figure S39. UV-Vis spectra from stability assays

Figure S40. Correlation between cytotoxic activity of the complexes and $\log D_{7.4}$.

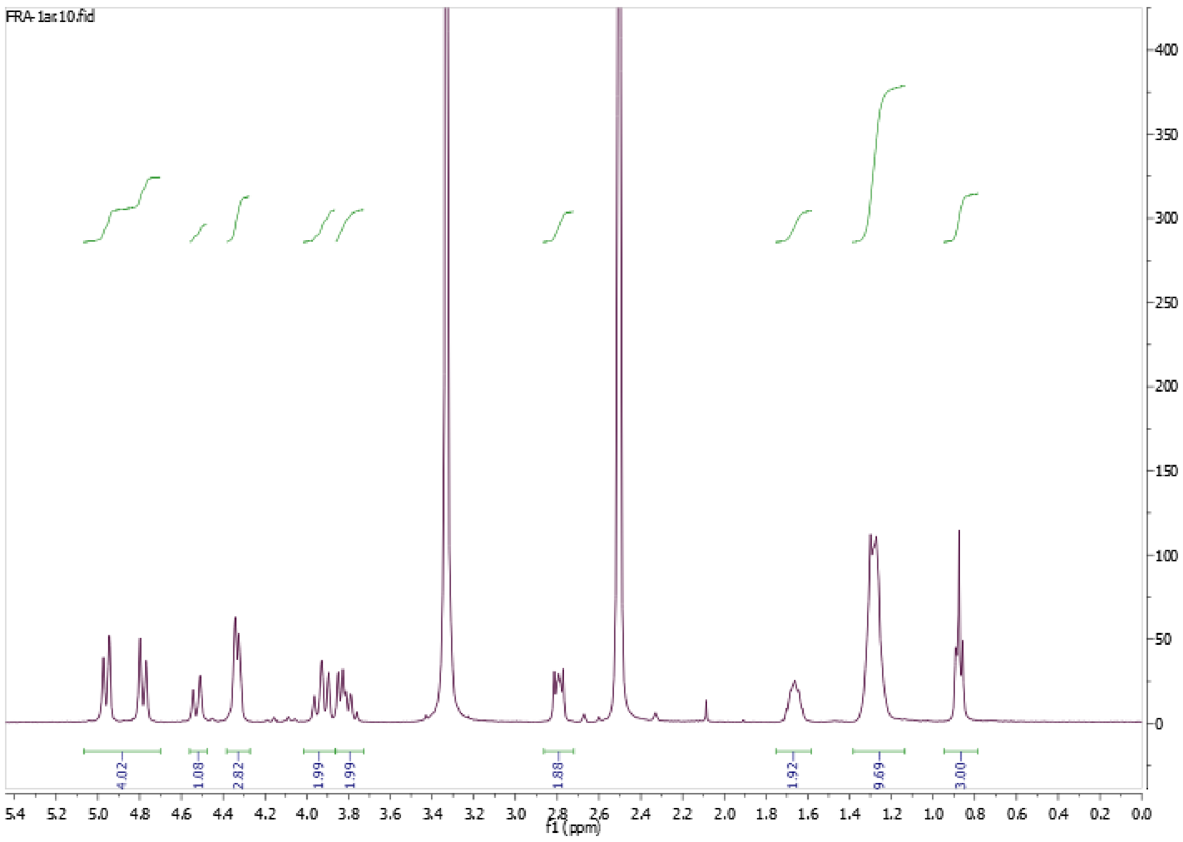

Figure S1. ^{1}H NMR spectrum of [PTA-CH_2-$(CH_2)_6$-CH_3]I (**La**)

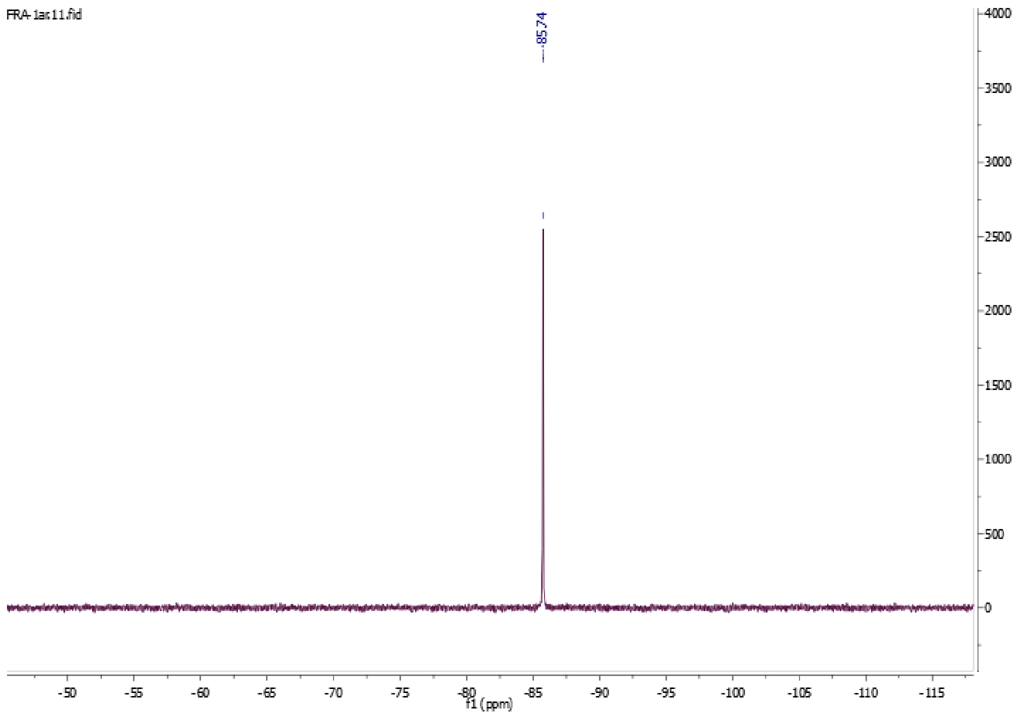

Figure S2. $^{31}P\{^{1}H\}$ NMR spectrum of [PTA-CH_2-$(CH_2)_6$-CH_3]I (**La**)

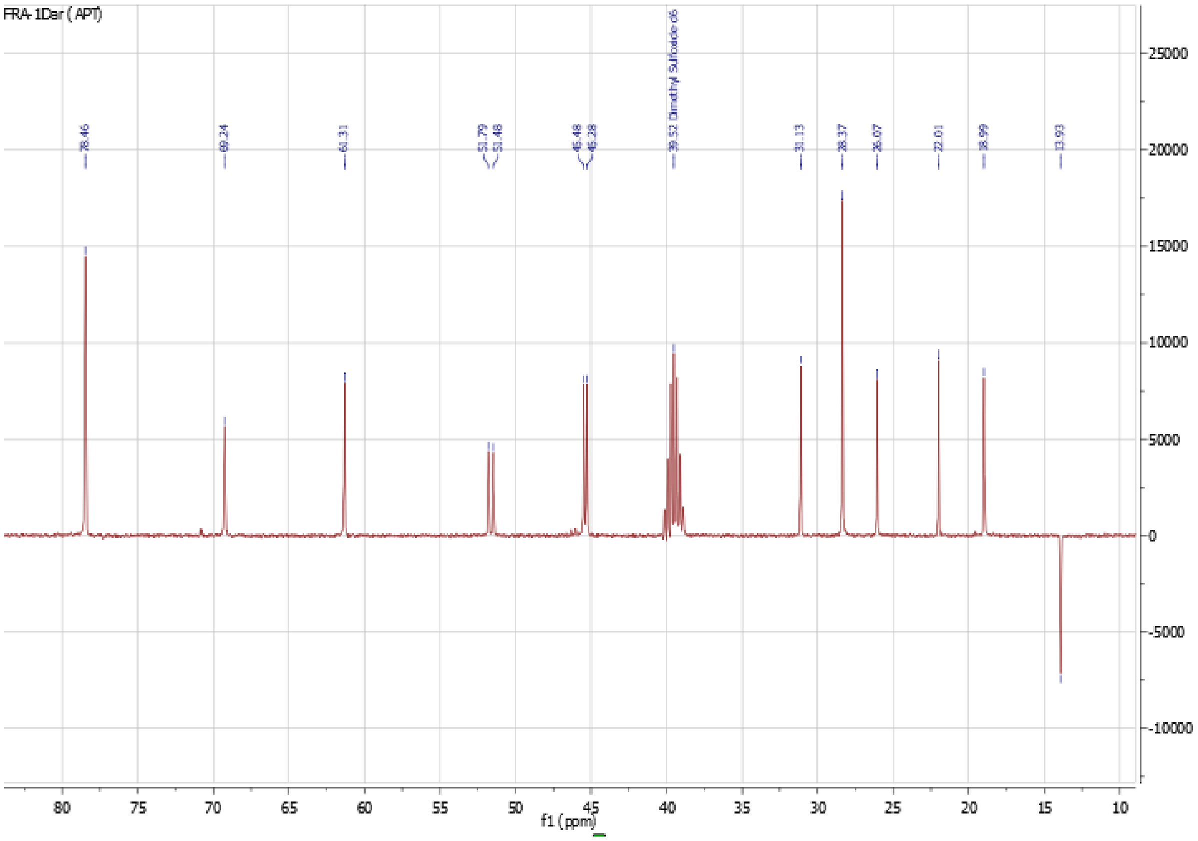

Figure S3. ^{13}C {^{1}H}-APT spectrum of [PTA-CH_2-$(CH_2)_6$-CH_3]I (**La**) in dmso-d_6

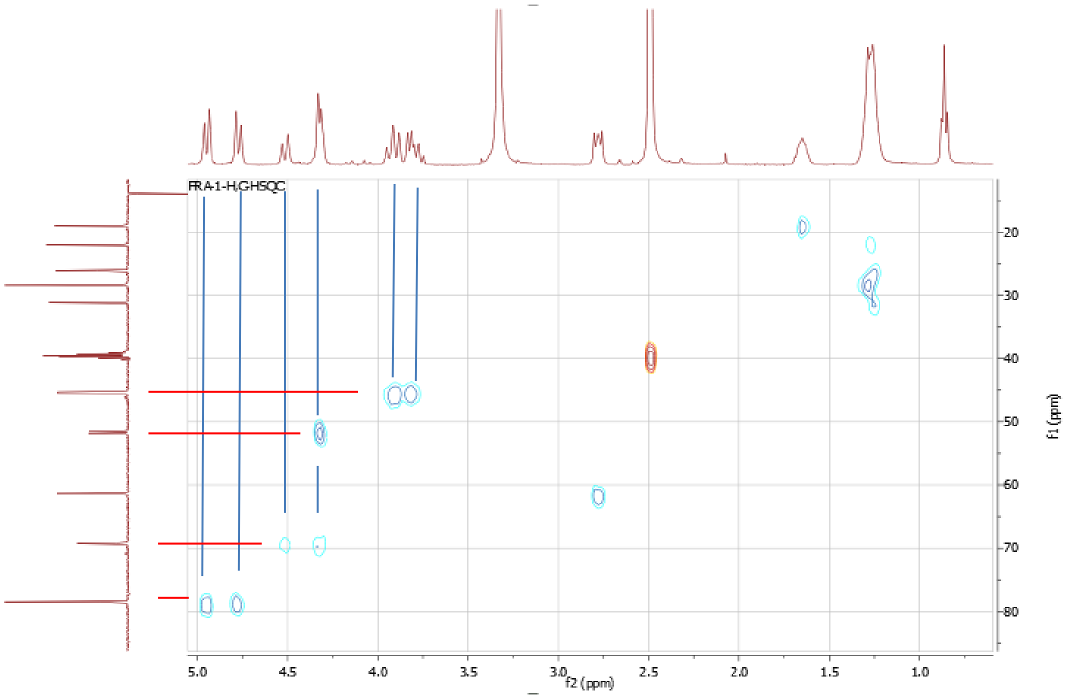

Figure S4. C,H-HSQC experiment of [PTA-CH_2-$(CH_2)_6$-CH_3]I (**La**) in dmso-d_6

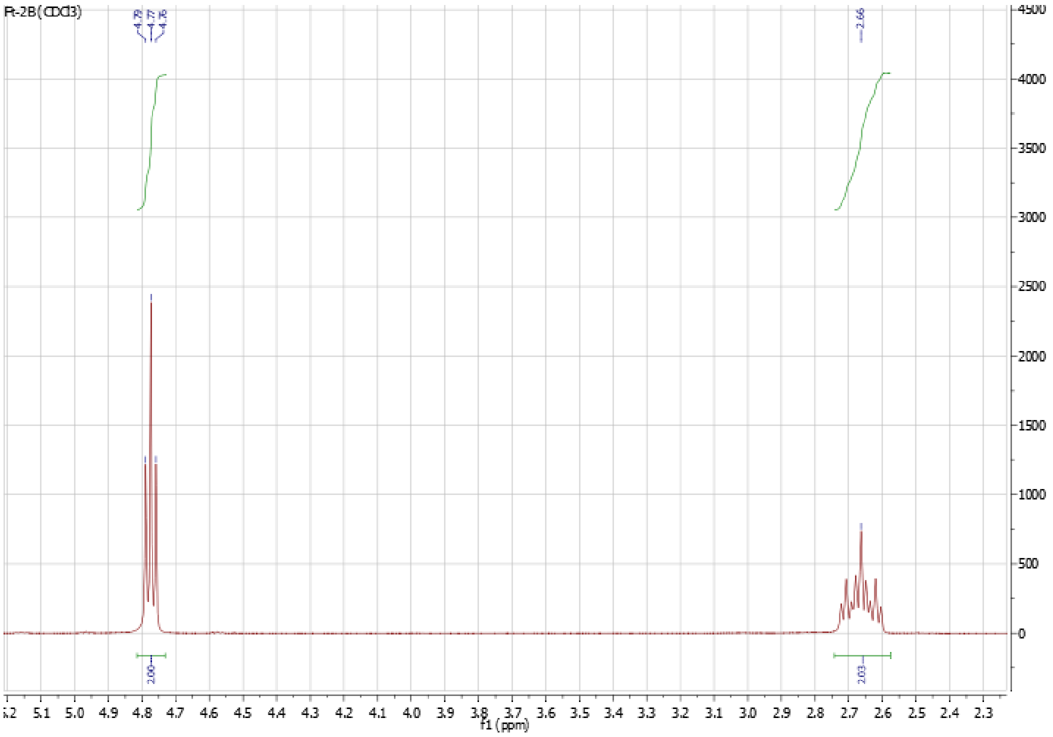

Figure S5. ^{1}H NMR spectrum of $TfOCH_2CH_2(CF_2)_5CF_3$ in $CDCl_3$

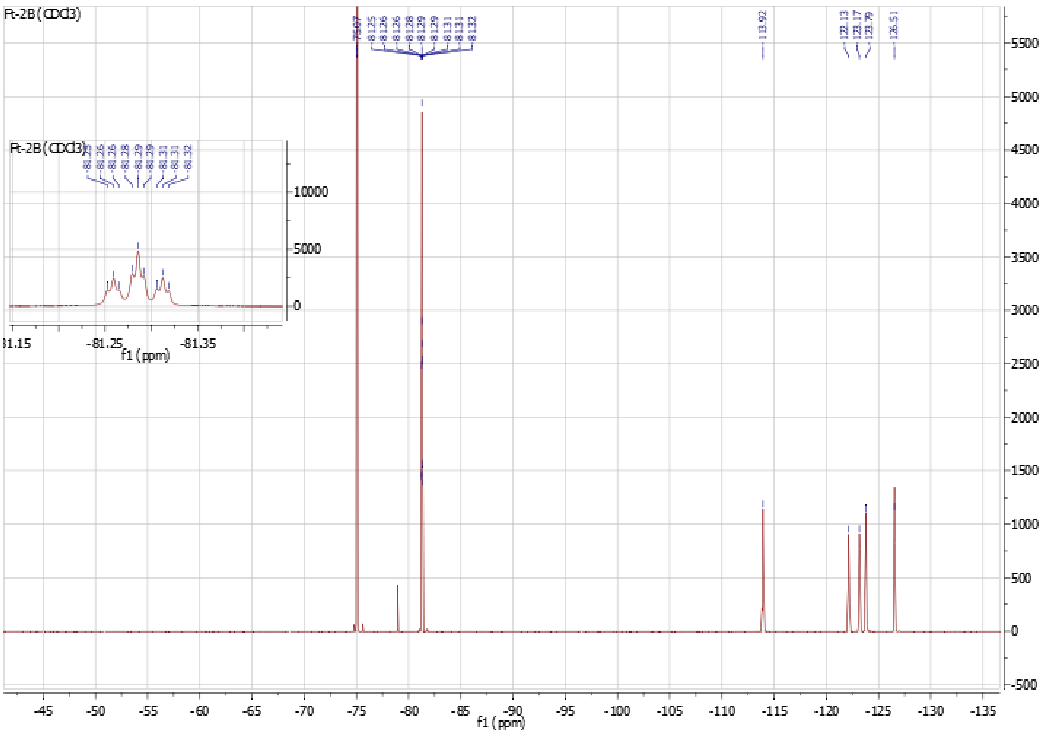

Figure S6. ^{19}F {^{1}H} NMR spectrum of $TfOCH_2CH_2(CF_2)_5CF_3$ in $CDCl_3$

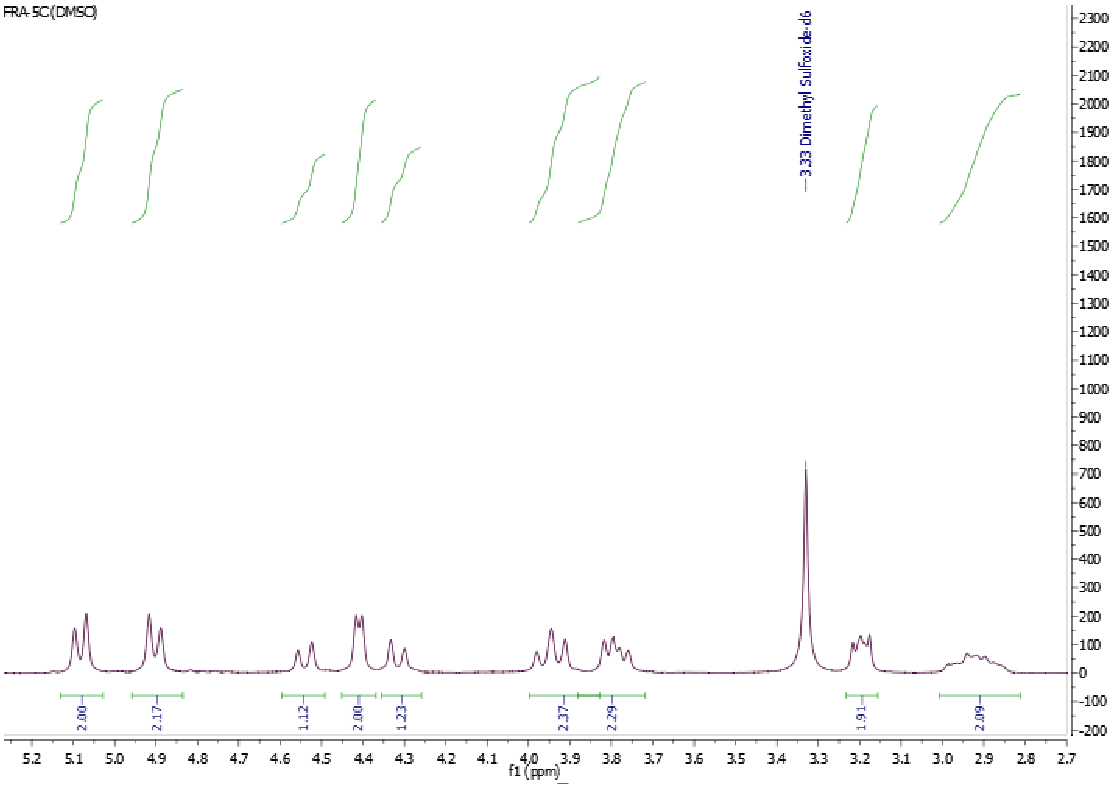

Figure S7. ^{1}H NMR spectrum of [PTA-$CH_2CH_2(CF_2)_5CF_3$]TfO (**Lb**) in dmso-d_6

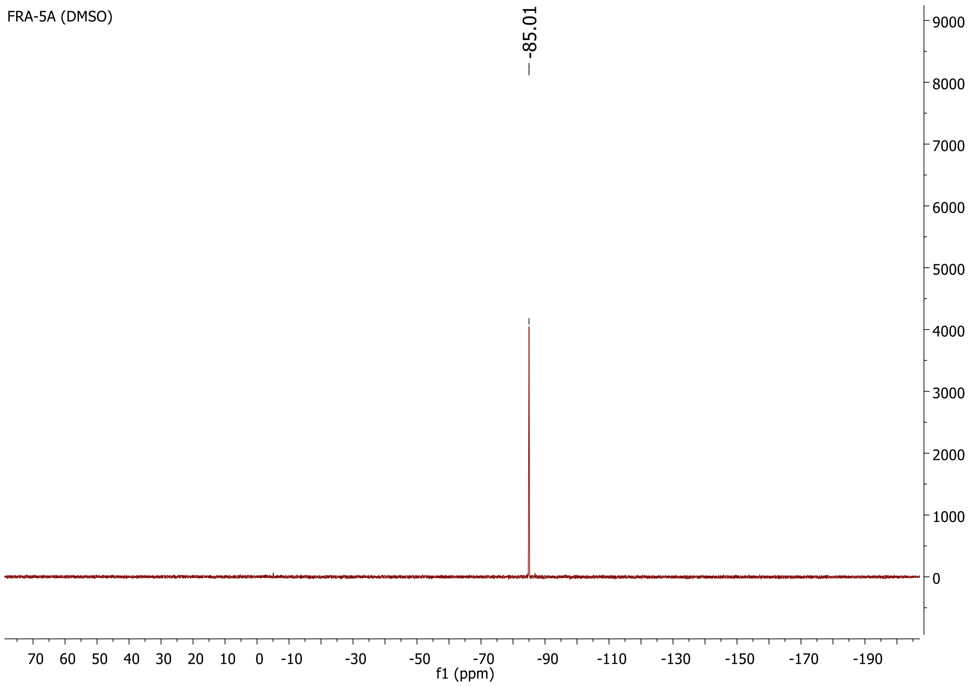

Figure S8. $^{31}P\{^{1}H\}$ NMR spectrum of [PTA-$CH_2CH_2(CF_2)_5CF_3$]TfO (**Lb**) in dmso-d_6

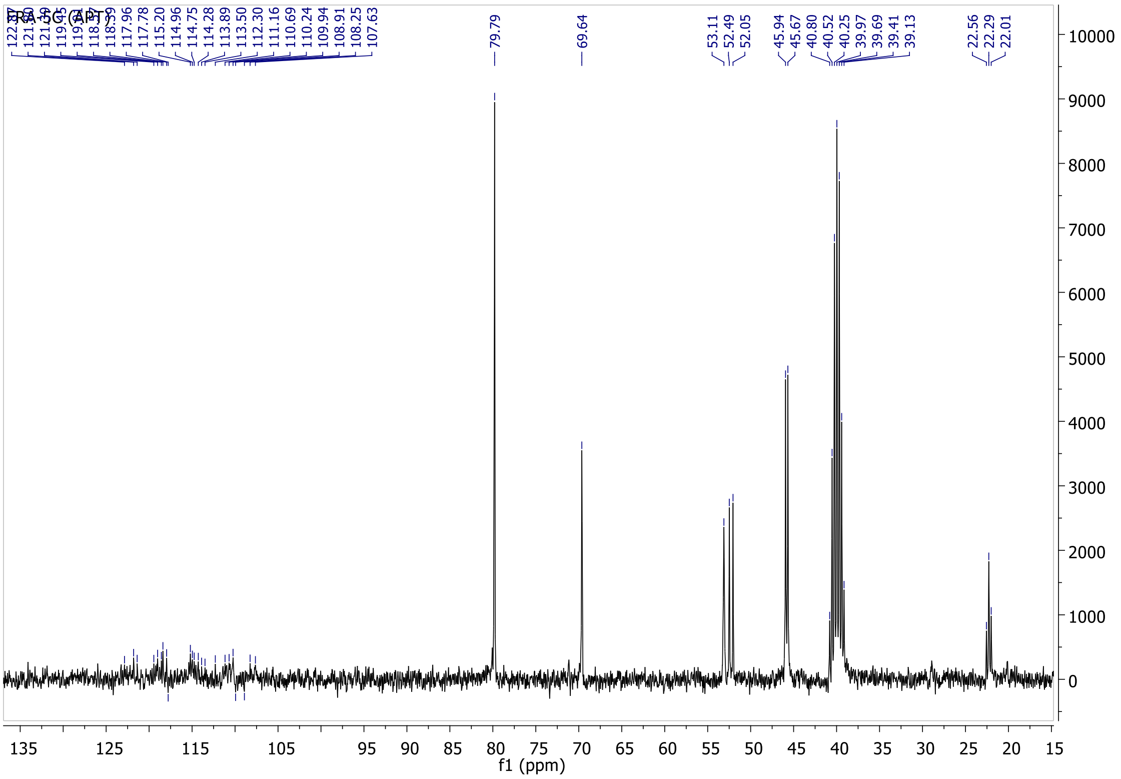

Figure S9. $^{13}C\ \{^{1}H\}$ NMR spectrum of [PTA-$CH_2CH_2(CF_2)_5CF_3$]TfO (**Lb**) in dmso-d_6

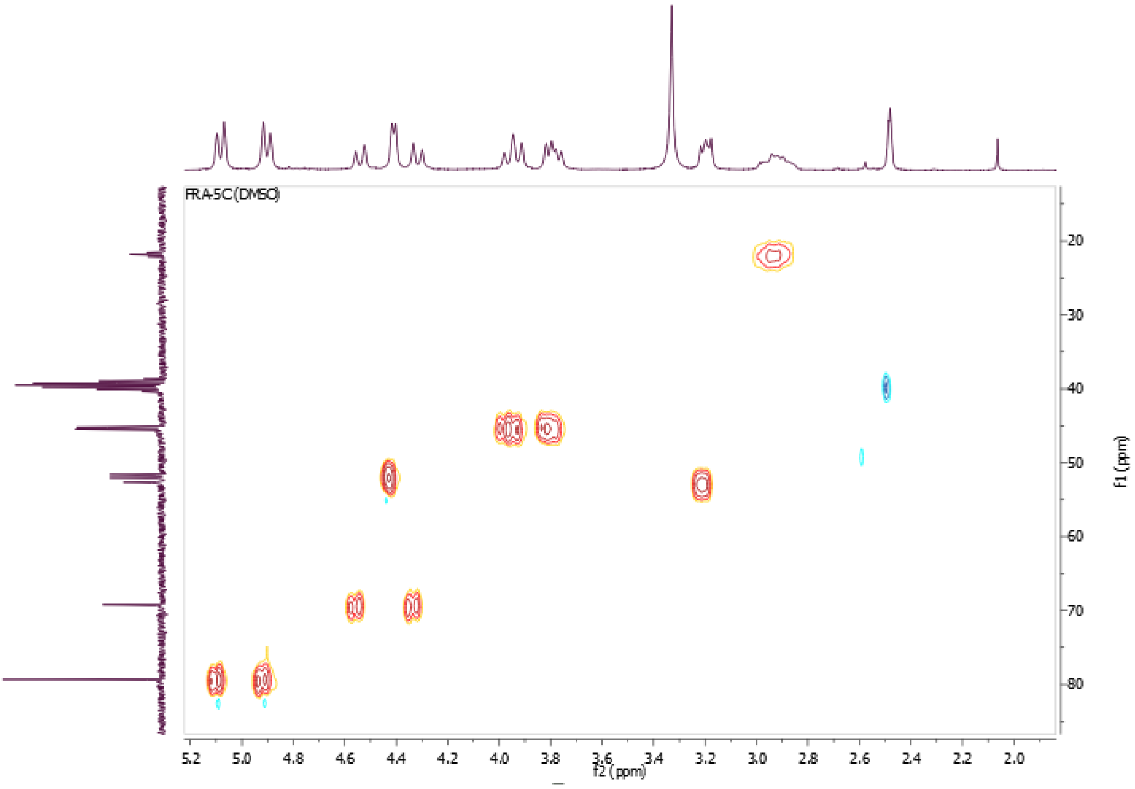

Figure S10. C,H-HSQC experiment of [PTA-$CH_2CH_2(CF_2)_5CF_3$]TfO (**Lb**) in dmso-d_6

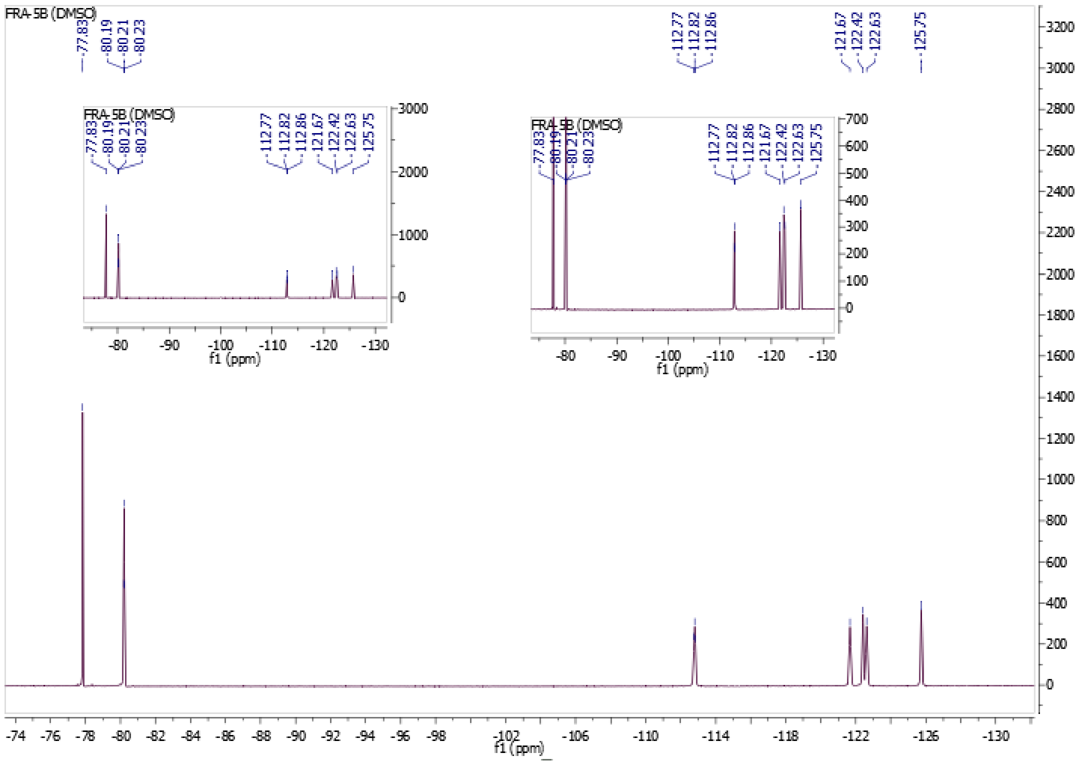

Figure S11. $^{19}F\ \{^{1}H\}$ NMR spectrum of [PTA-$CH_2CH_2(CF_2)_5CF_3$]TfO (**Lb**) in dmso-d_6

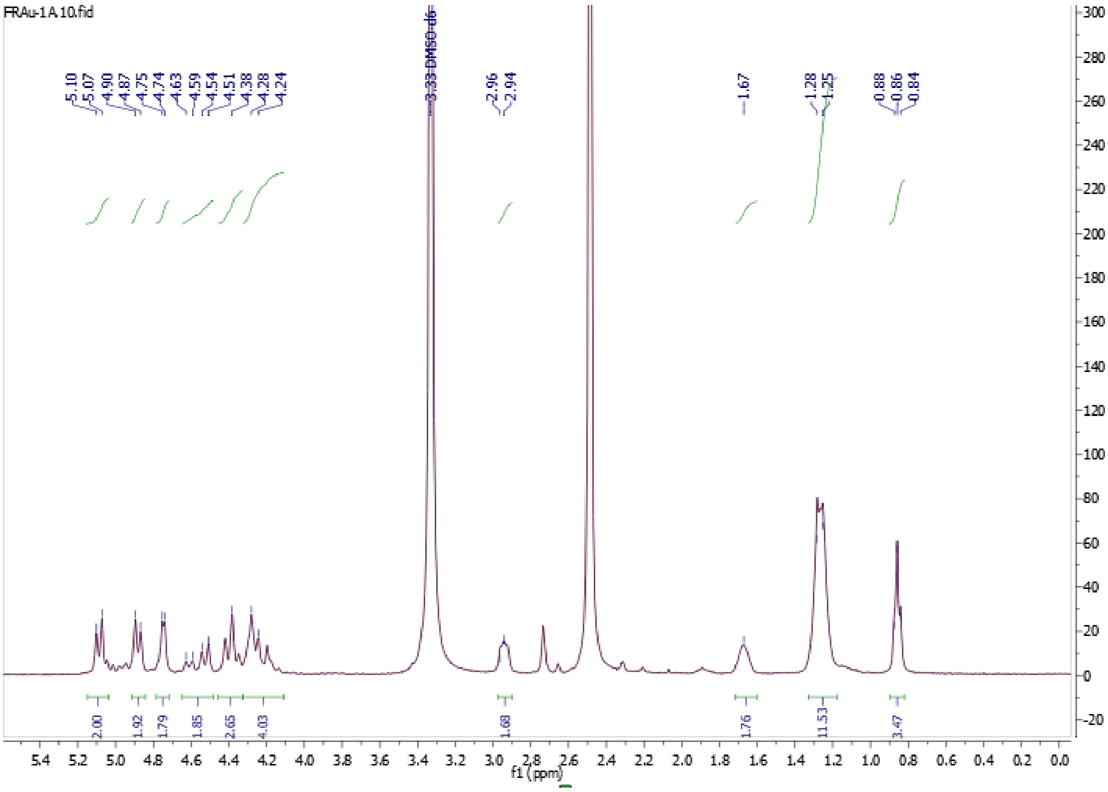

Figure S12. ^{1}H NMR spectrum of [AuCl(PTA-$CH_2(CH_2)_6CH_3$)]I (**1a**) in dmso-d_6

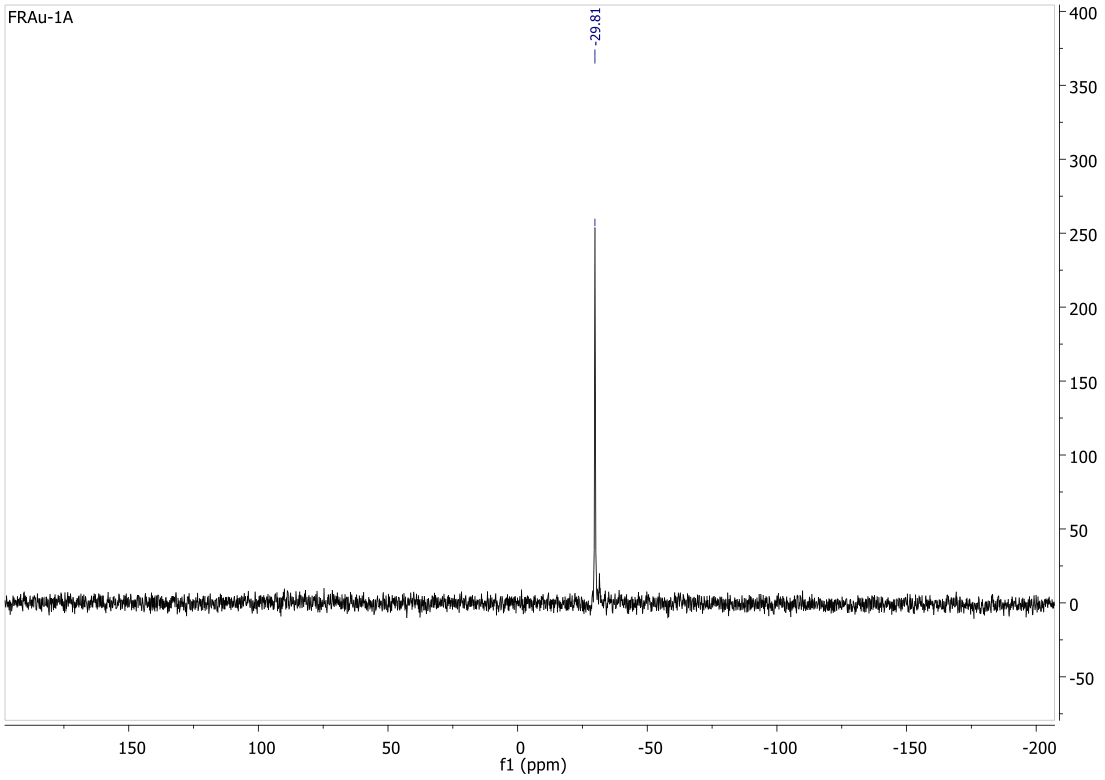

Figure S13. $^{31}P\{^{1}H\}$ NMR spectrum of [AuCl(PTA-$CH_2(CH_2)_6CH_3$)]I (**1a**) in dmso-d_6

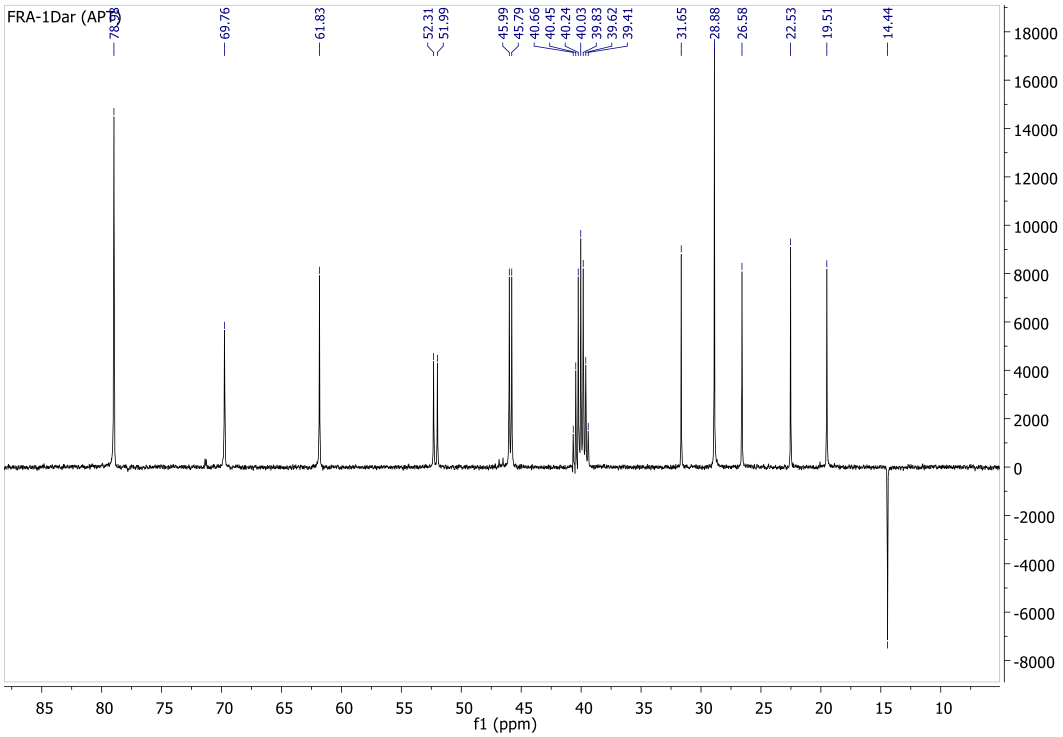

Figure S14. $^{13}C\ \{^{1}H\}$**-APT spectrum of [AuCl(PTA-**$CH_2(CH_2)_6CH_3$**)]I (1a) in dmso-**d_6

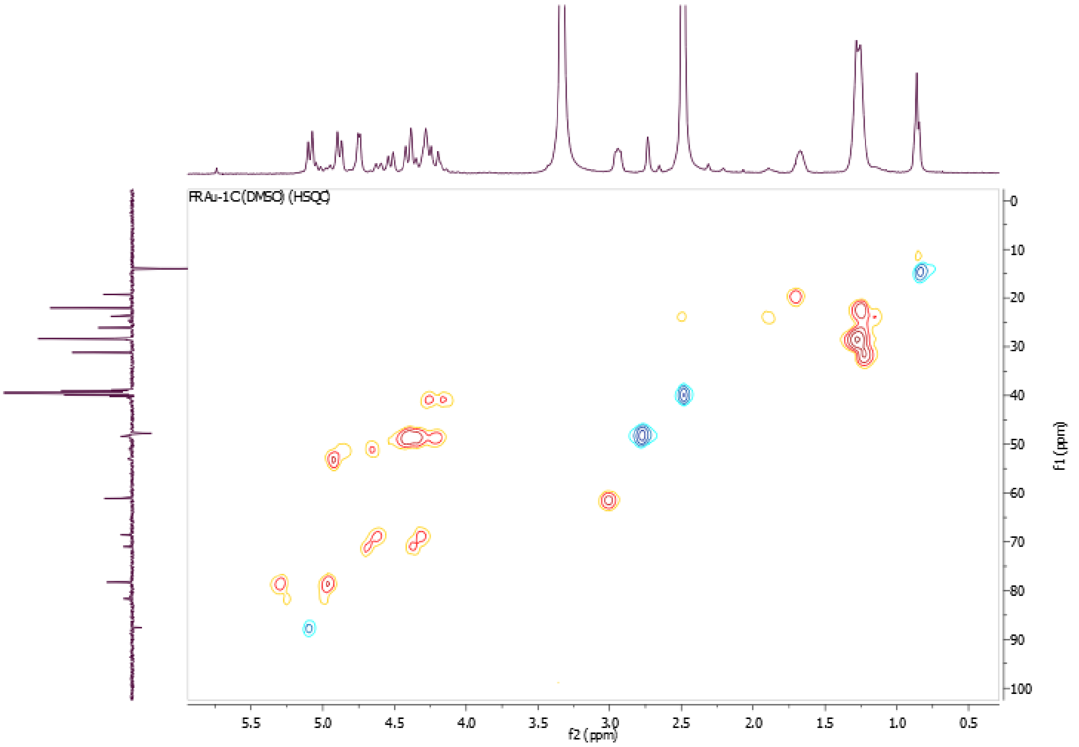

Figure S15. C,H-HSQC experiment of [AuCl(PTA-$CH_2(CH_2)_6CH_3$)]I (**1a**) in dmso-d_6

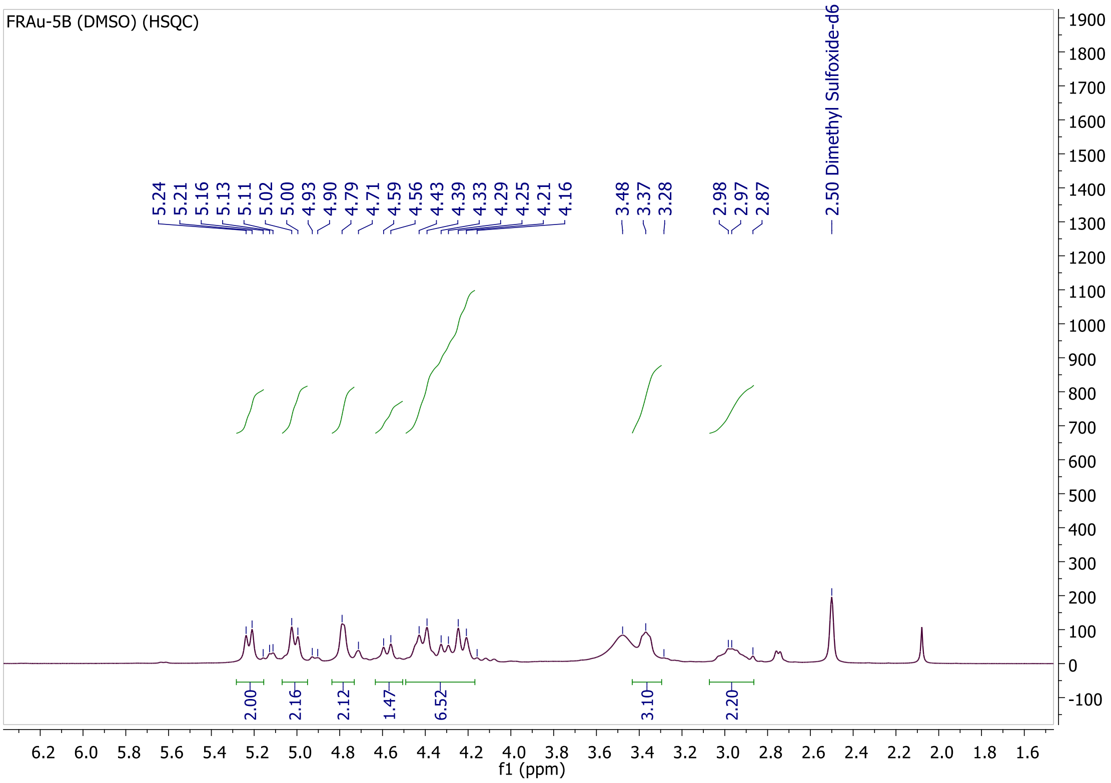

Figure S16 ^{1}H NMR spectrum of [AuCl(PTA-$CH_2CH_2(CF_2)_5CF_3$)]TfO (**1b**) in dmso-d_6

Figure 6.25 IR spectrum of compound (**4**)

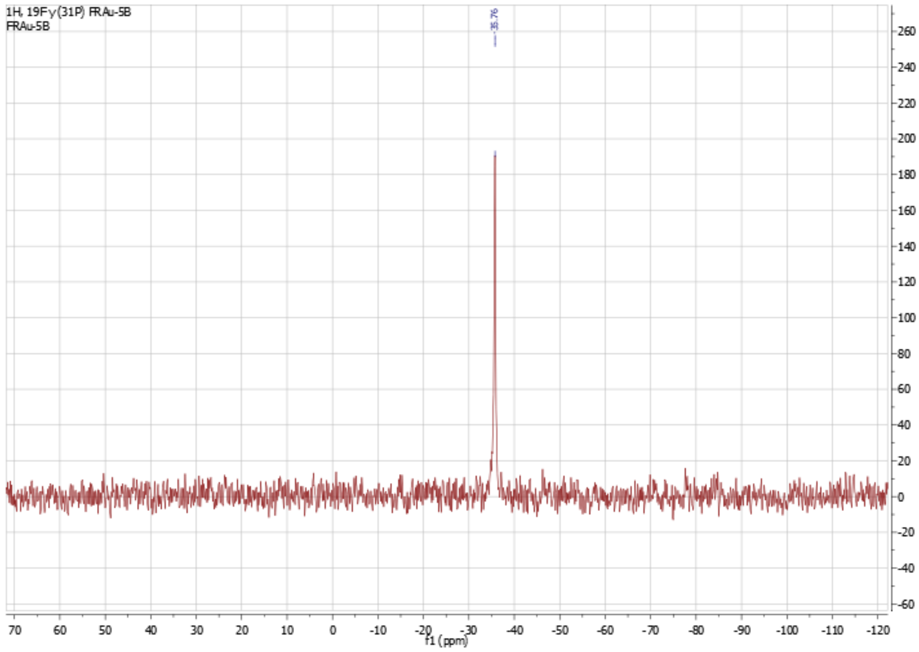

Figure S17. $^{31}P\{^{1}H\}$ NMR spectrum of [AuCl(PTA-$CH_2CH_2(CF_2)_5CF_3$)]TfO (**1b**) in dmso-d_6

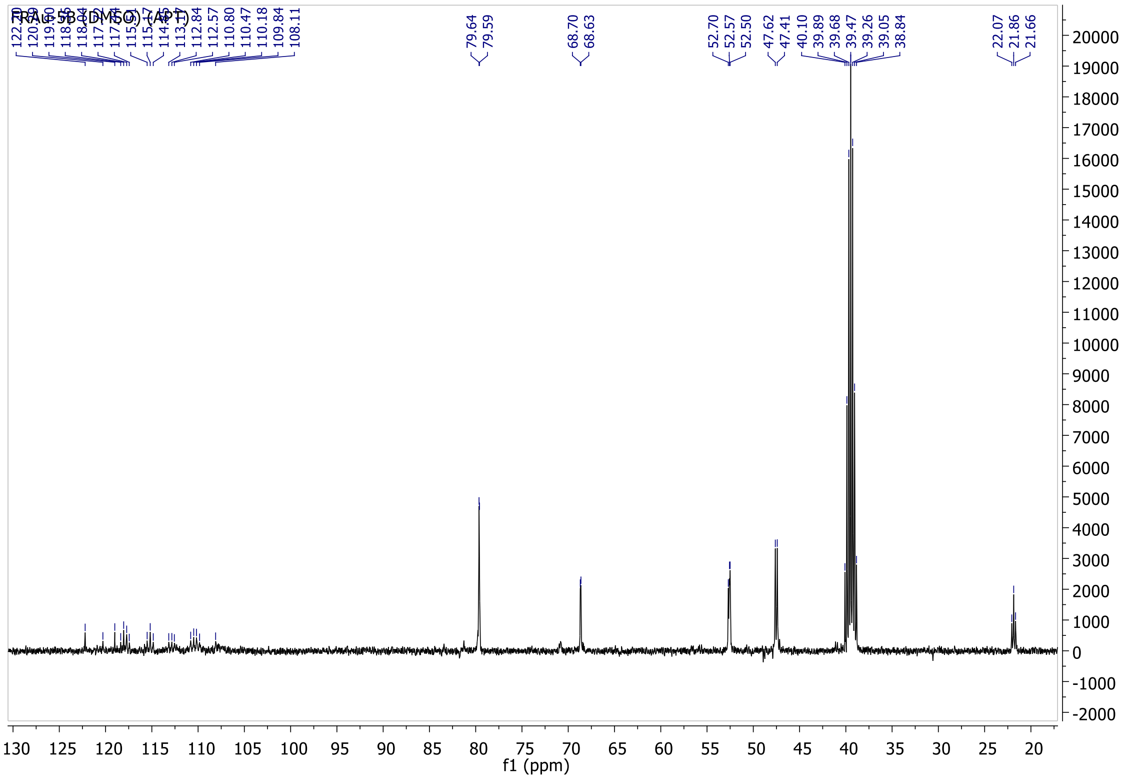

Figure S18. ^{13}C $\{^{1}H\}$ NMR spectrum of [AuCl(PTA-$CH_2CH_2(CF_2)_5CF_3$)]TfO (**1b**) in dmso-d_6

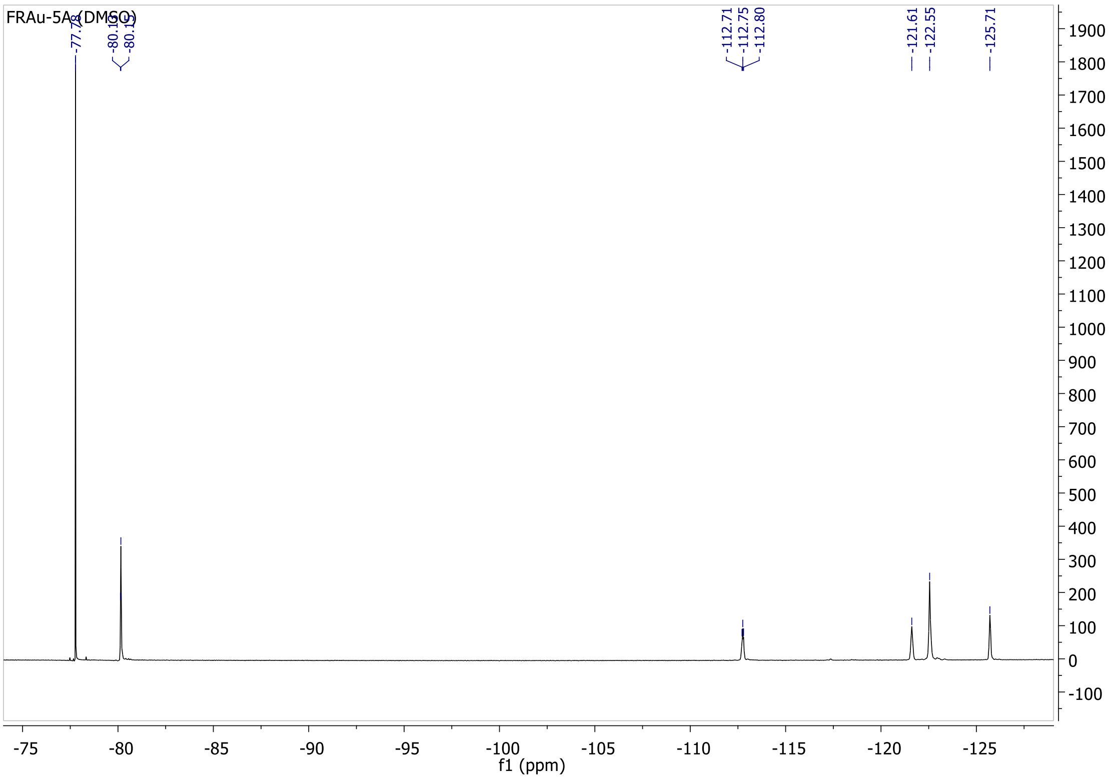

Figure S19. ^{19}F {^{1}H} NMR spectrum of [AuCl(PTA-$CH_2CH_2(CF_2)_5CF_3$)]TfO (**1b**) in dmso-d_6

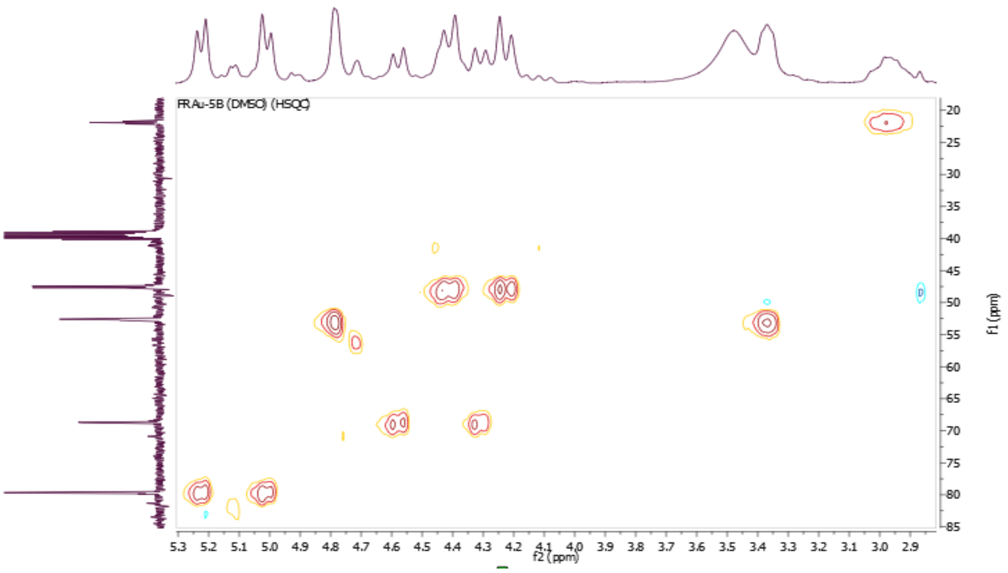

Figure S20. C,H-HSQC experiment of [AuCl(PTA-$CH_2CH_2(CF_2)_5CF_3$)]TfO (**1b**) in dmso-d_6

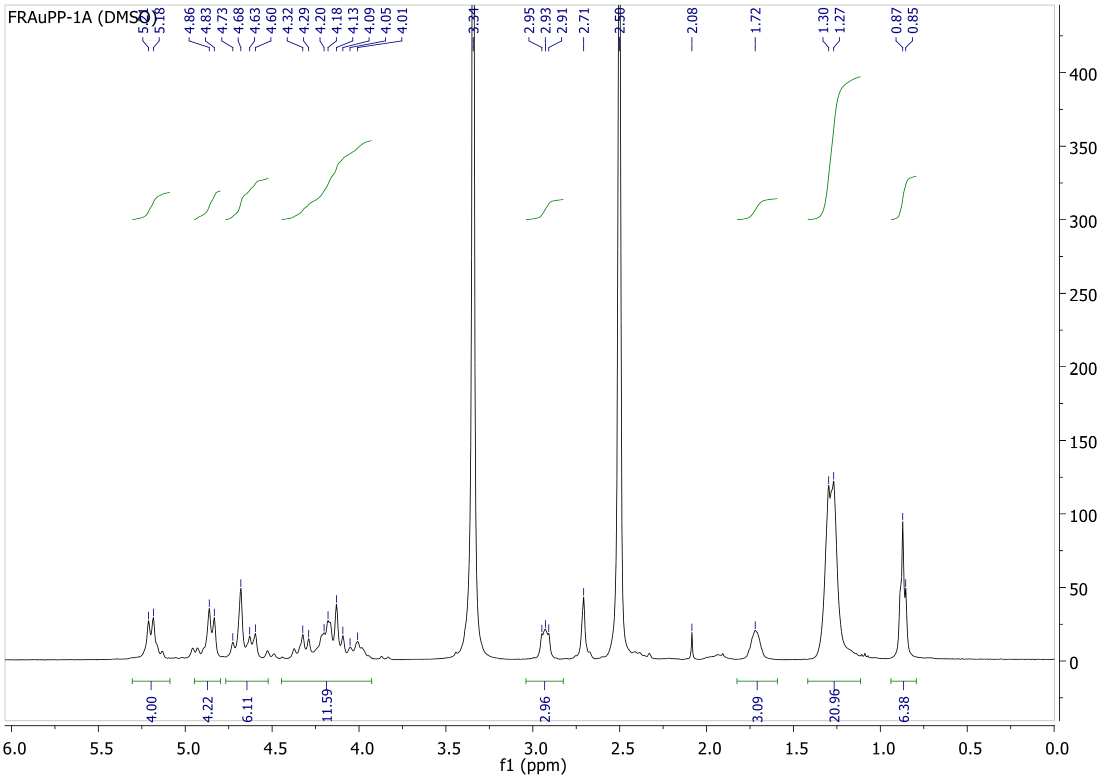

Figure S21 ^{1}H NMR spectrum of $[AuCl(PTA\text{-}CH_2(CH_2)_6CH_3)_2]I_2$ (**2a**) in dmso-d_6

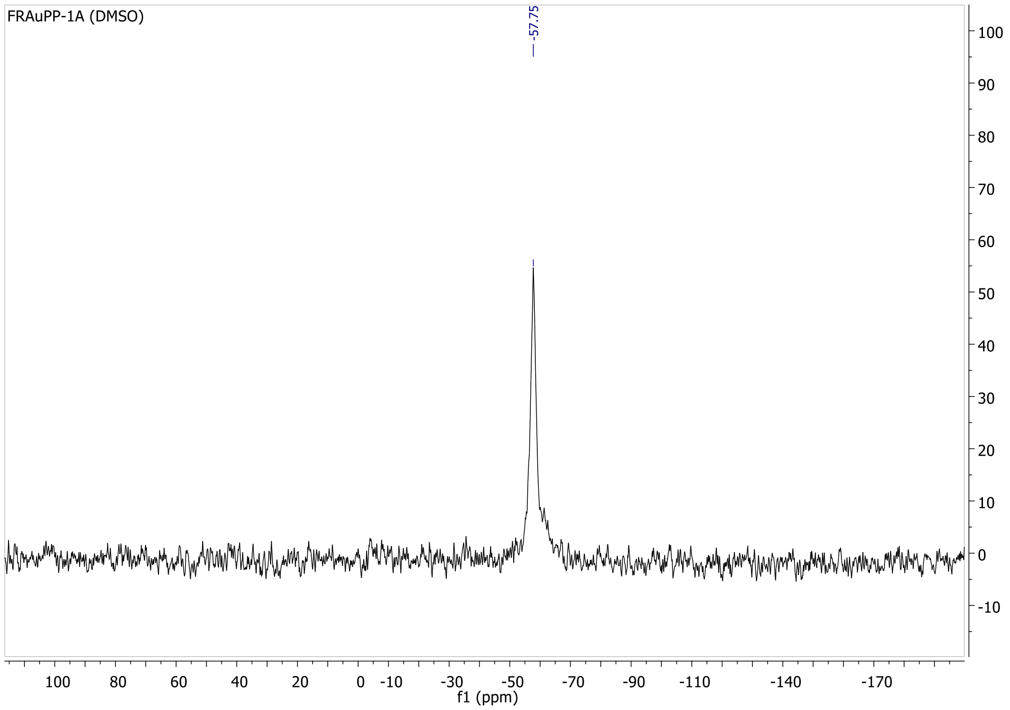

Figure S22. $^{31}P\{^{1}H\}$ NMR spectrum of (**2a**) in dmso-d_6

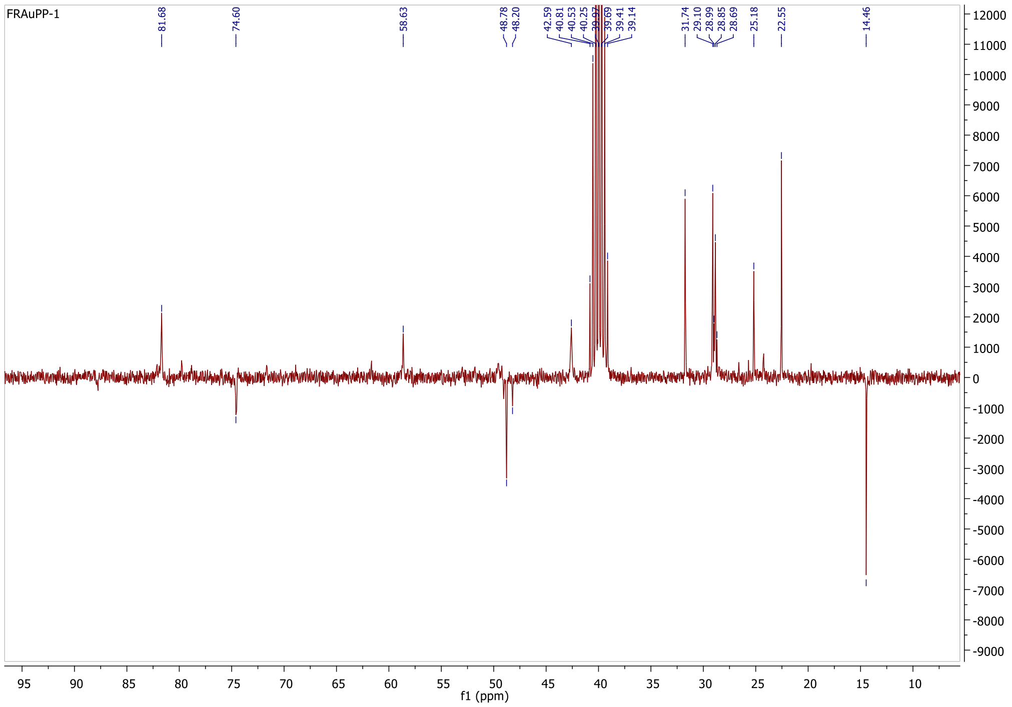

Figure S23. ^{13}C {^{1}H}-APT spectrum of (**2a**) in dmso-d_6

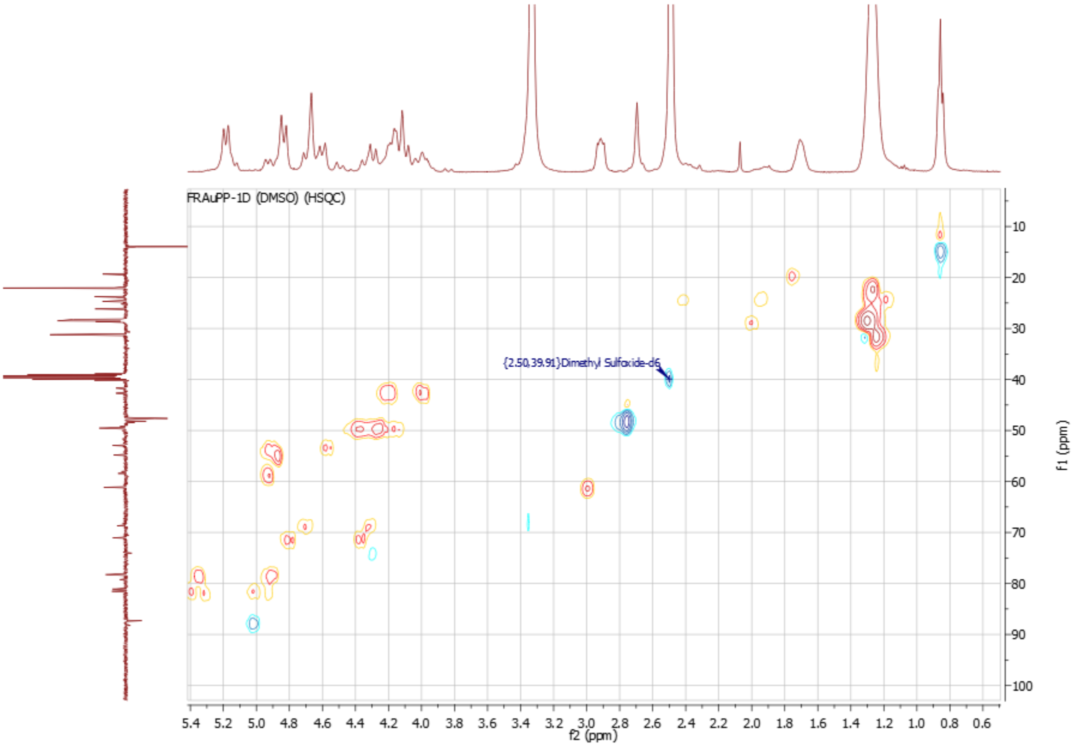

Figure S24. C,H-HSQC experiment of[AuCl(PTA-$CH_2(CH_2)_6CH_3)_2$]I_2 (**2a**) in dmso-d_6

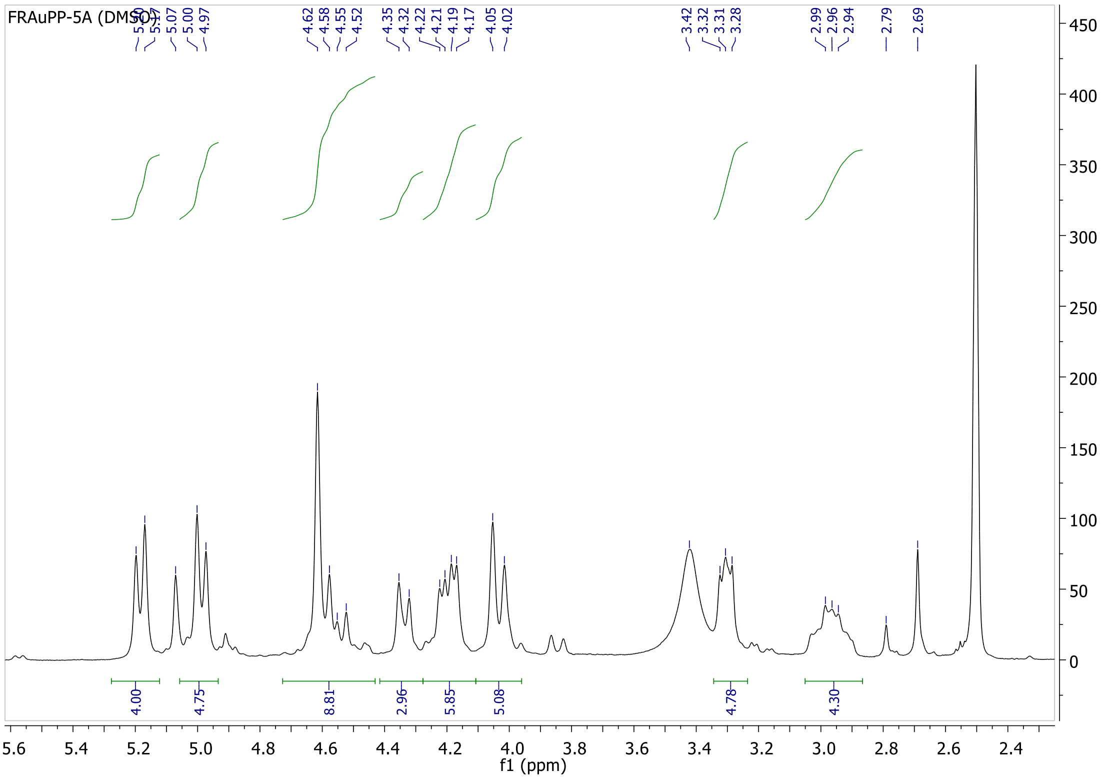

Figure S25. ^{1}H NMR spectrum of [AuCl(PTA-$CH_2CH_2(CF_2)_5CF_3$)$_2$](TfO)$_2$ (**2b**) in dmso-d_6

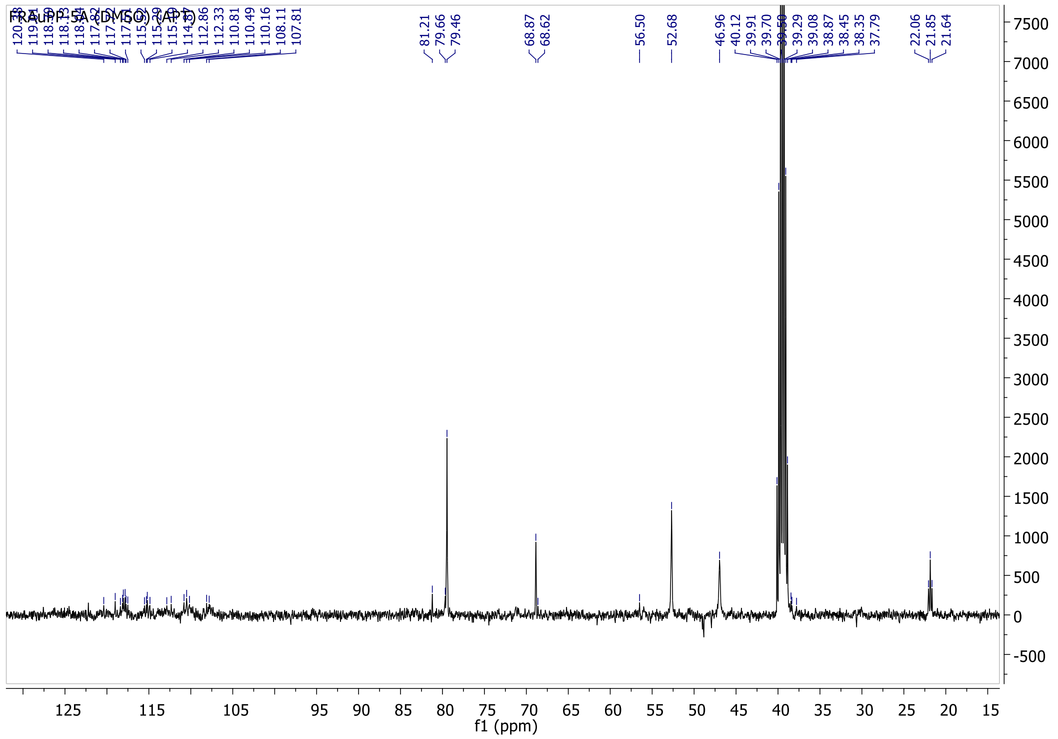

Figure S26. ^{13}C {^{1}H} NMR spectrum of [AuCl(PTA-$CH_2CH_2(CF_2)_5CF_3$)$_2$](TfO)$_2$ (**2b**) in dmso-d_6

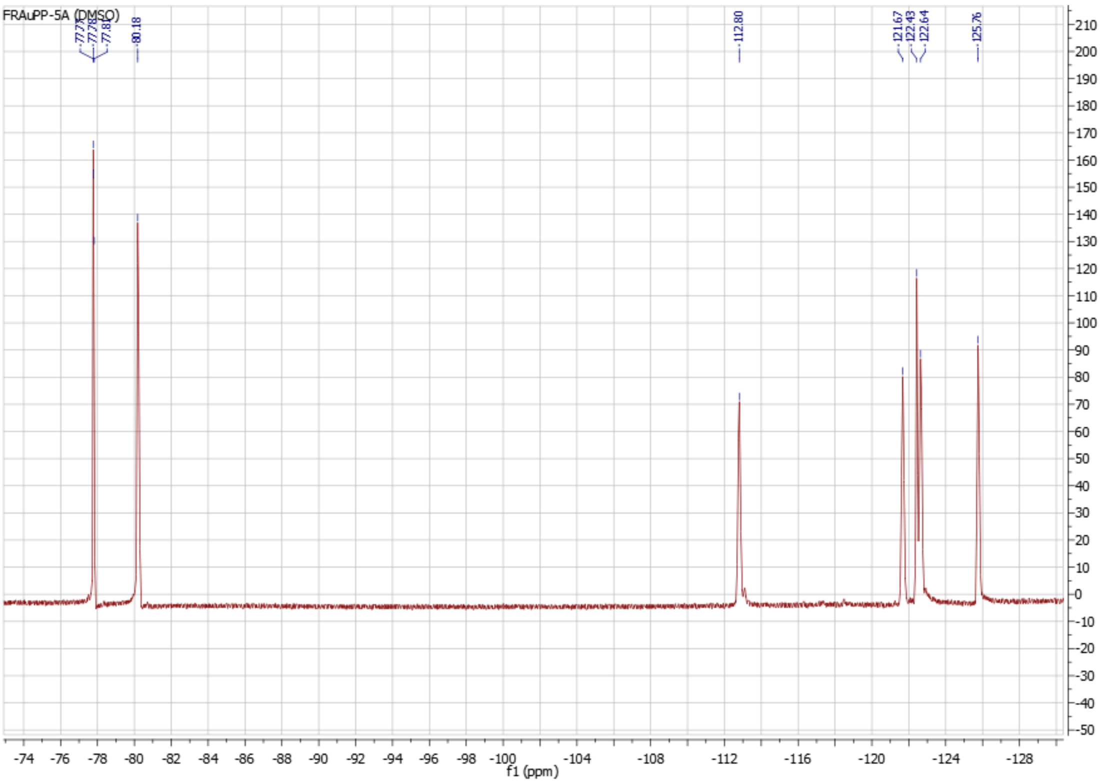

Figure S27. $^{19}F\{^{1}H\}$ NMR of [AuCl(PTA-$CH_2CH_2(CF_2)_5CF_3)_2$]$(TfO)_2$ (**2b**) in dmso-d_6

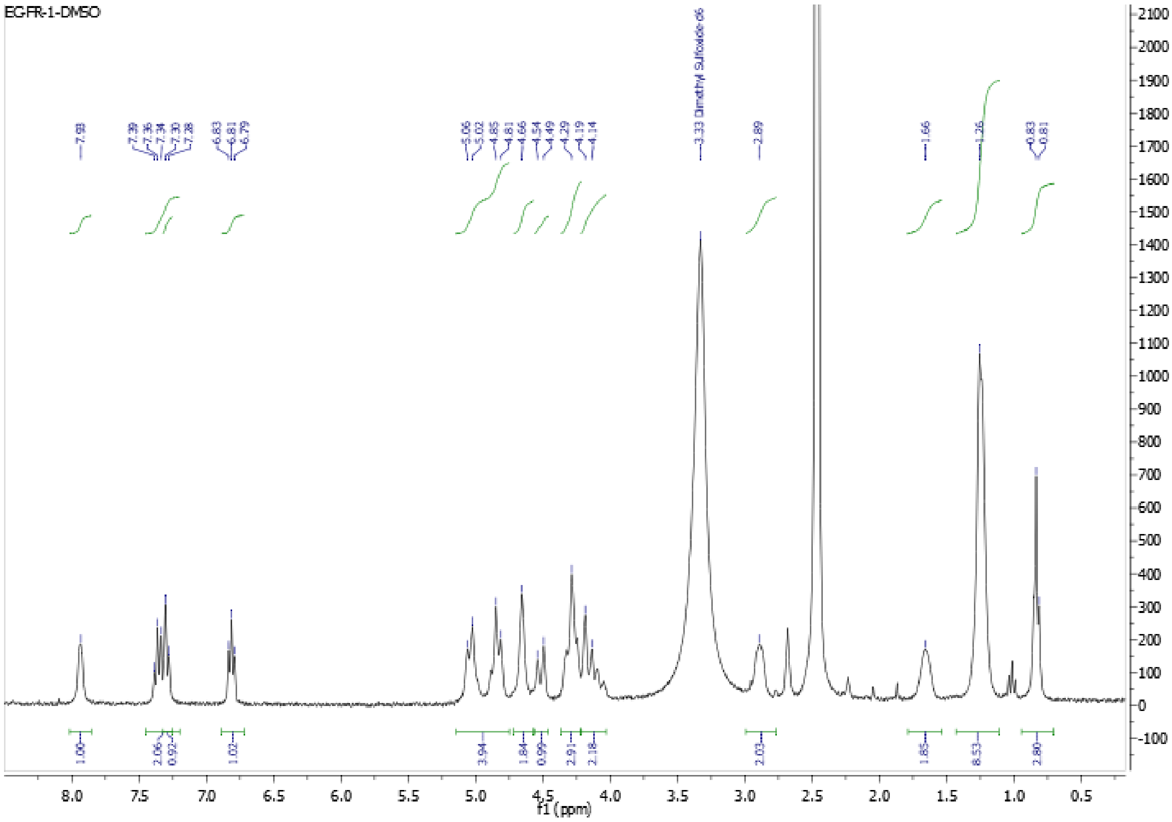

Figure S28. ^{1}H NMR spectrum of [Au(C_5H_5NS)(PTA-$CH_2(CH_2)_6CH_3$)]I (**3a**) in dmso-d_6

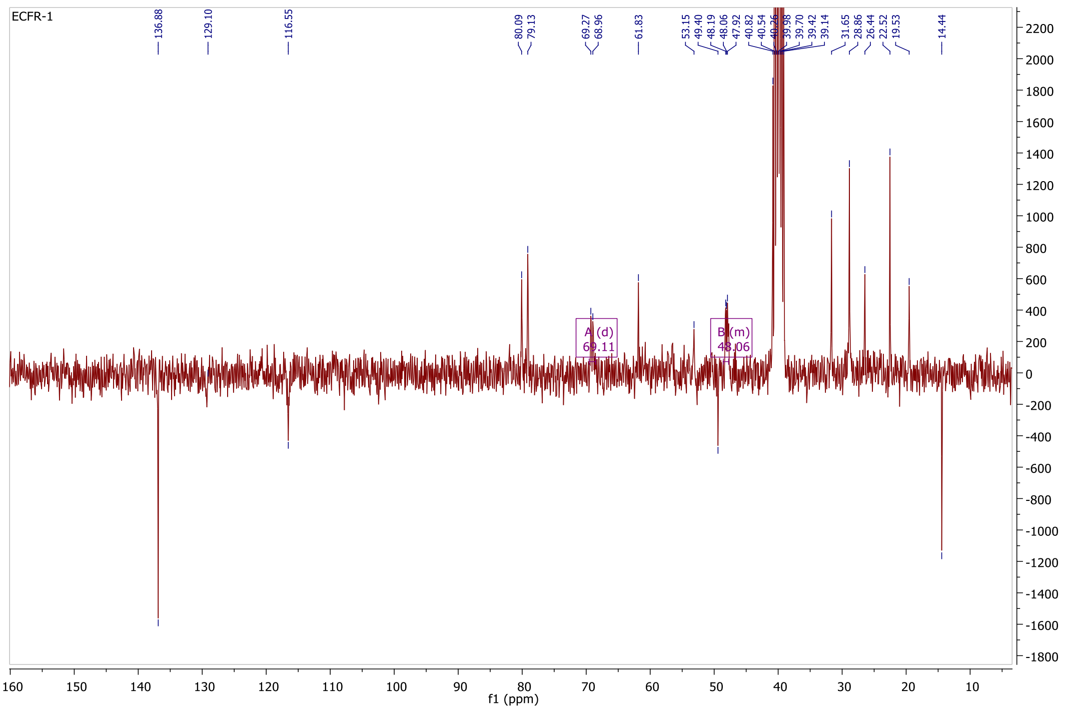

Figure S29. $^{13}C\ \{^{1}H\}$-apt NMR spectrum of $[Au(C_5H_5NS)(PTA\text{-}CH_2(CH_2)_6CH_3)]I$ (**3a**) in dmso-d_6

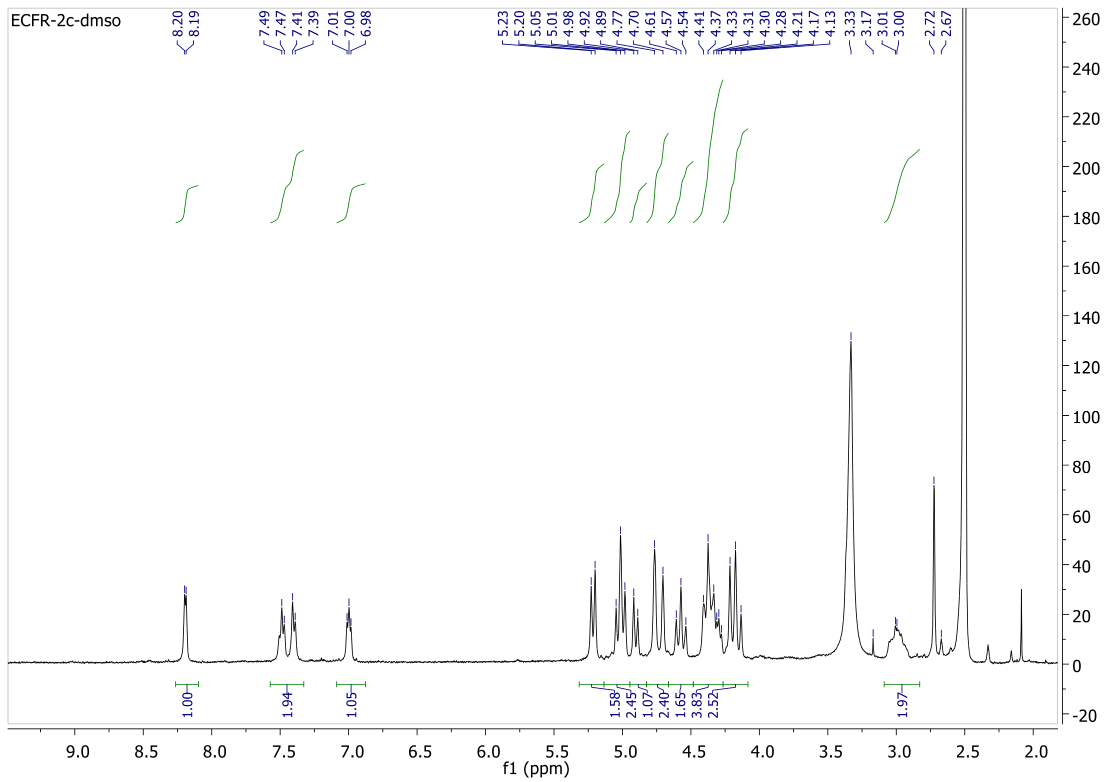

Figure S30. ^{1}H NMR spectrum of $[Au(C_5H_5NS)(PTA\text{-}CH_2CH_2(CF_2)_5CF_3)]TfO$ (**3b**) in dmso-d_6

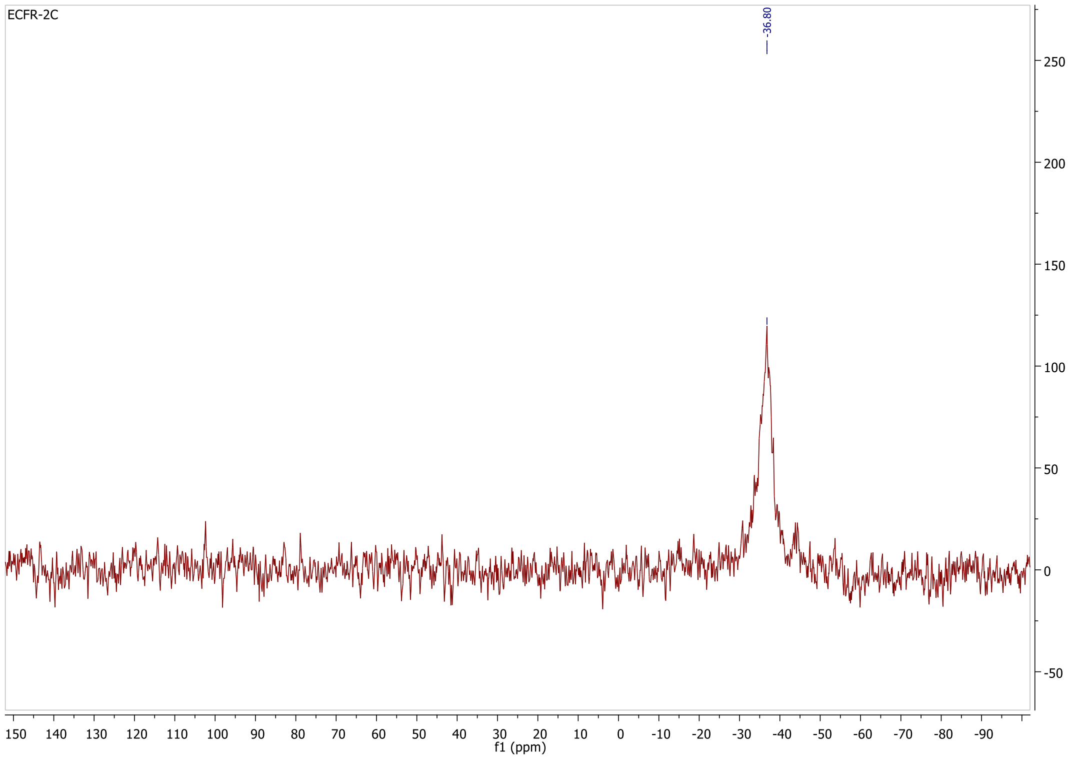

Figure S36. $^{31}P\{^{1}H\}$ NMR spectrum of **3b**

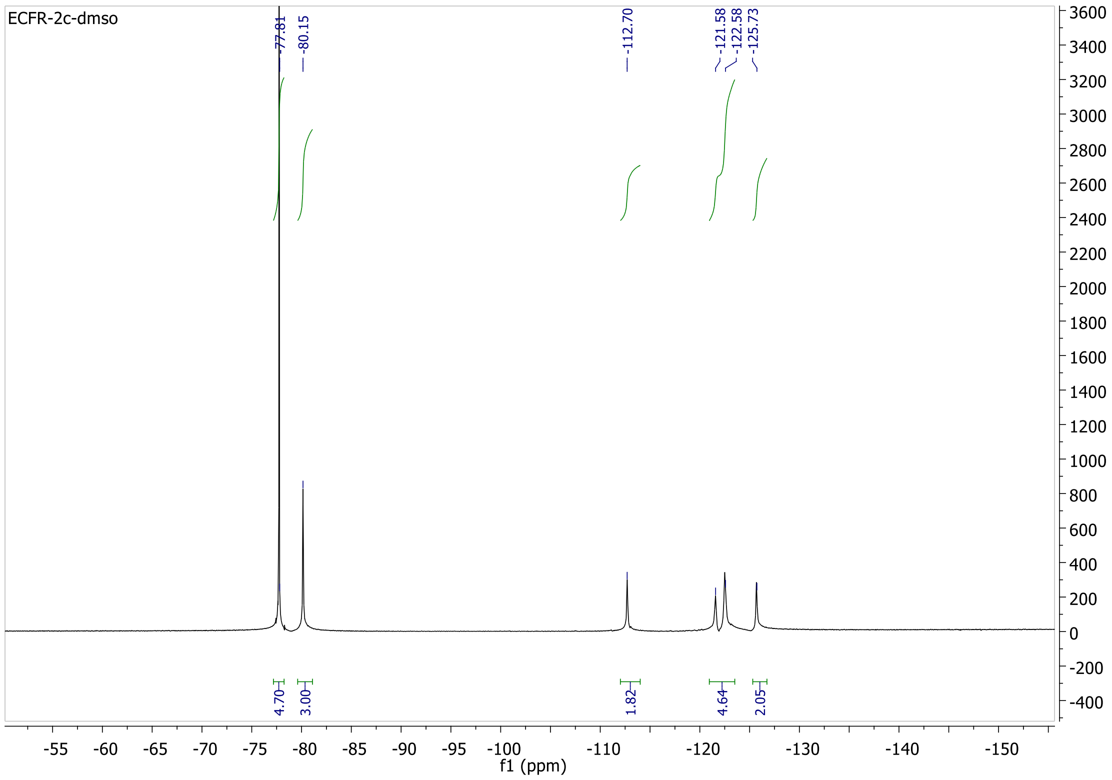

Figure S37. $^{19}F\{^{1}H\}$ NMR spectrum of **3b**

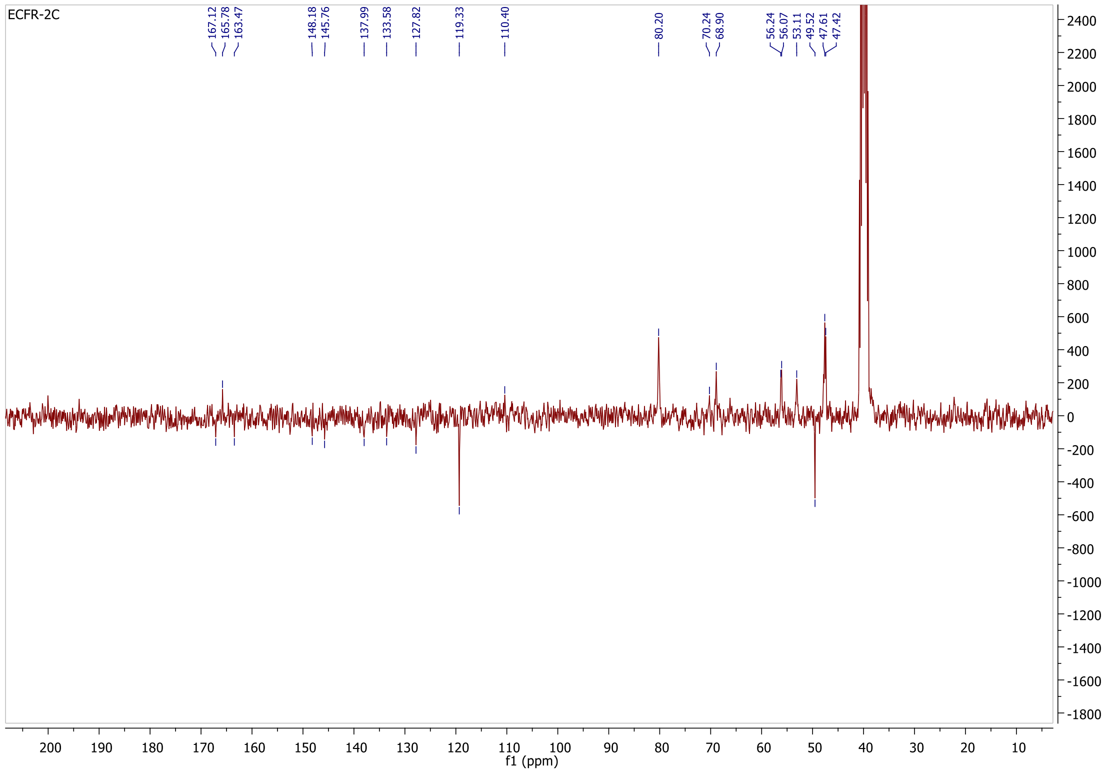

Figure S38. ^{13}C {^{1}H}-apt NMR spectrum of **3b**

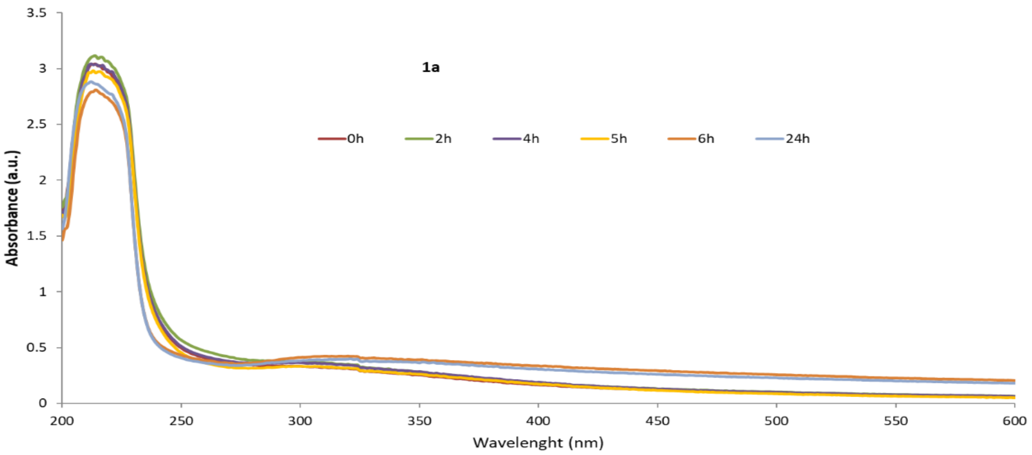

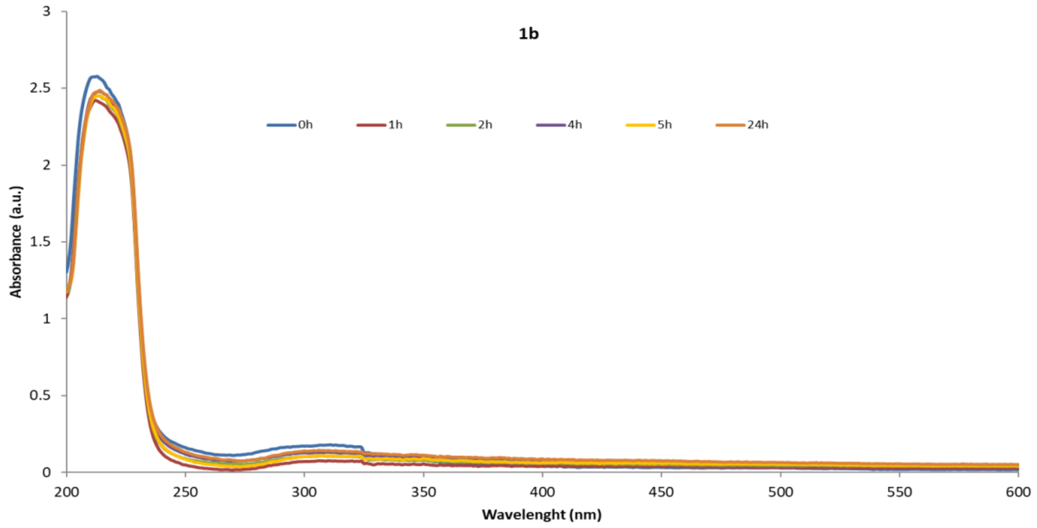
1b
0h
1h
2h
4h
5h
24h
Absorbance (a.u.)
Wavelenght (nm)

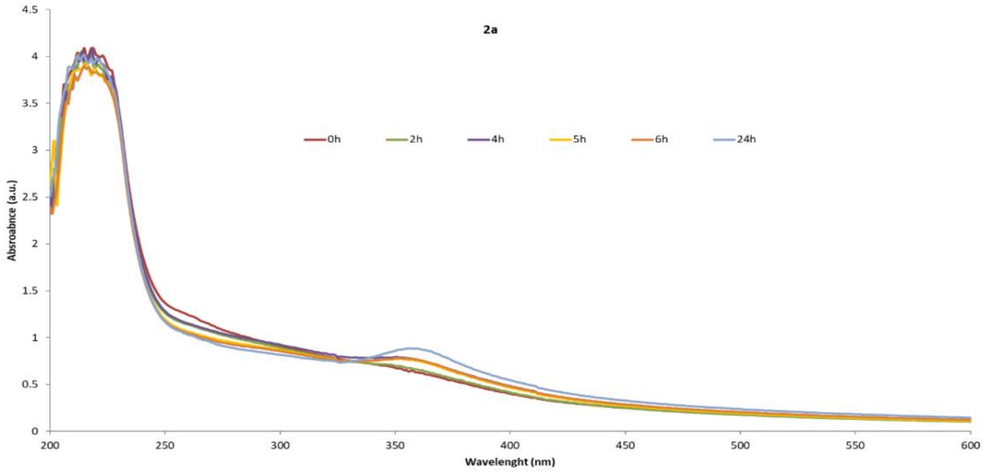
2a
0h
2h
4h
5h
6h
24h
Absroabnce (a.u.)
Wavelenght (nm)

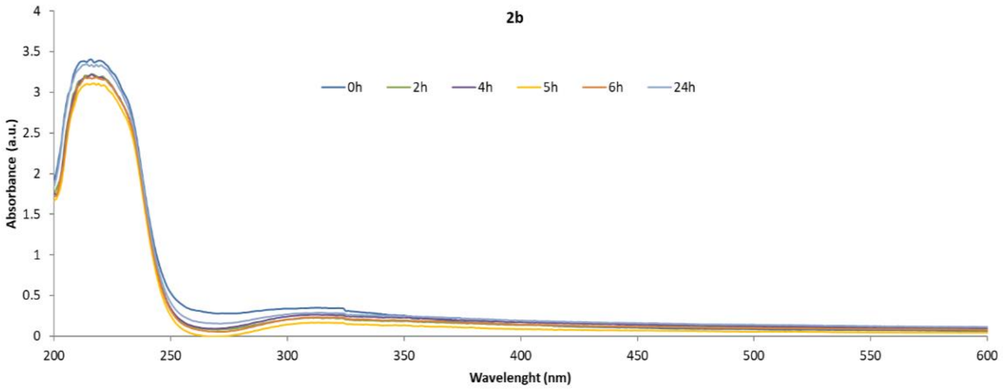
2b
0h
2h
4h
5h
6h
24h
Absorbance (a.u.)
Wavelenght (nm)

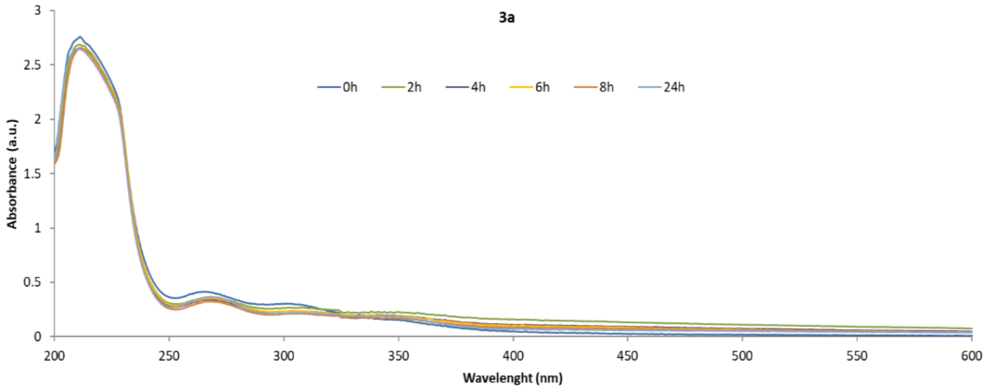

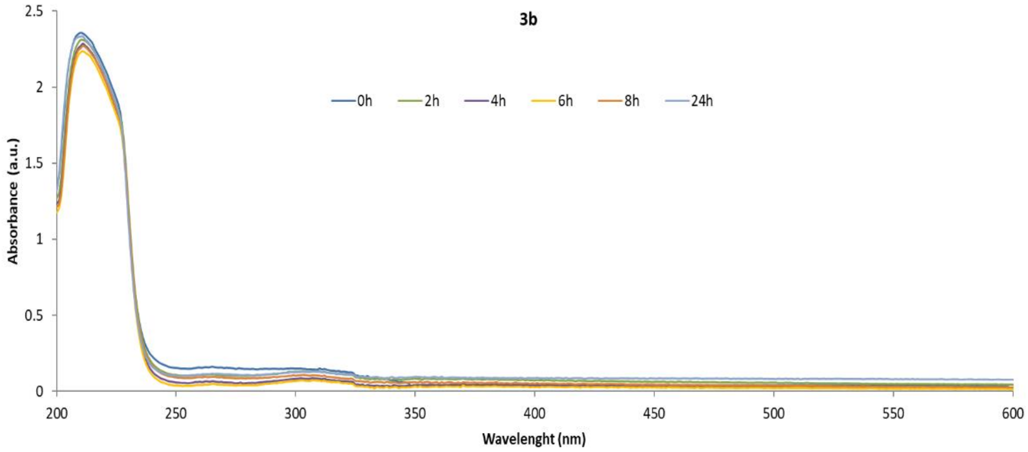

Figure S39. UV-Vis spectra of gold complexes recorded at different times for 24 h to test their stability under physiological conditions. [complex] = 30 µM diluted in PBS at 37 ºC.

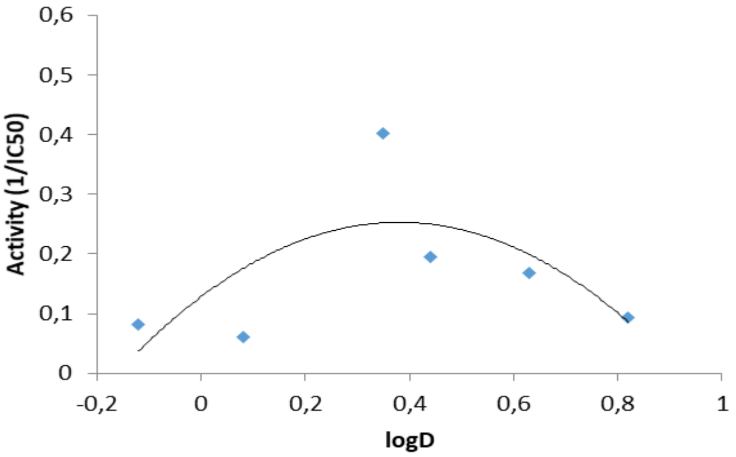

Figure S40. Correlation between cytotoxic activity of the complexes and log$D_{7.4}$. The activities are calculated as the inverse of IC50 in Caco-2/PD7 cells.
